# Enhancing Professionalism Online (Netiquette) in Medical Schools: A Systematic Scoping Review

**DOI:** 10.1177/23821205241255268

**Published:** 2025-02-24

**Authors:** Donovan Kai Wei Ng, Jonathan Zhen Liang, Ruth Si Man Wong, Vijayprasanth Raveendran, Gillian Li Gek Phua, Warren Fong, Crystal Lim, Jamie Xuelian Zhou, Lalit Kumar Radha Krishna

**Affiliations:** 1 Yong Loo Lin School of Medicine, National University Singapore, Singapore; 2 Division of Supportive & Palliative Care, 68751 National Cancer Centre Singapore, Singapore; 3 Duke-NUS Medical School, Singapore; 4 Lien Centre for Palliative Care, Duke-NUS Medical School, National University of Singapore, Singapore; 5 Department of Rheumatology and Immunology, 37581 Singapore General Hospital, Singapore; 6 Medical Social Services, 37581 Singapore General Hospital, Singapore; 7 Division of Cancer Education, 68751 National Cancer Centre Singapore, Singapore; 8 Palliative Care Institute Liverpool, Academic Palliative & End of Life Care Centre, 4591 University of Liverpool, Liverpool, UK; 9 Centre for Biomedical Ethics, National University of Singapore, Singapore; 10 The Palliative Care Centre for Excellence in Research and Education, Singapore; 11 Health Data Science, 4591 University of Liverpool, Liverpool, UK

**Keywords:** medical education and training, medical schools, ethics, medical students, netiquette, online etiquette, online professionalism, online learning, information technology

## Abstract

**Background:**

The relaxing of COVID-19 pandemic restrictions has not seen the return to previous in-person teaching formats. As blended training continues to be used, there is emphasis on the need to better appreciate the expectations, etiquette, and professional code of conduct (“netiquette”) surrounding online learning, especially in light of evidence that poor online professionalism compromises learning and clinical practice.

**Objectives:**

This review seeks to map regnant netiquette guidelines in medical schools that will inform and provide preliminary recommendations for a clinically relevant framework.

**Design:**

This study is a systematic scoping review (SSR).

**Methods:**

Krishna's Systematic Evidence-Based Approach (SEBA)'s Constructivist ontological and Relativist epistemological lens was used to guide this SSR. The SEBA process involves 6 stages, including the *Systematic Approach, Split Approach, Jigsaw Perspective, Funneling, Analysis of evidence-based and non-data-driven literature*, and *Synthesis of the SSR in SEBA*.

**Results:**

In total, 7941 abstracts were reviewed, 198 full text articles were evaluated, and 83 articles were included. The analysis of the results revealed 4 key domains: (1) current guidelines, (2) manifestations, (3) contributing factors, and (4) implications. This SSR in SEBA highlights variability and gaps in current guidelines and reveals the impact of sociocultural factors on breaches in netiquette. Unsurprisingly, contextual and clinical considerations shape the contributory factors impacting lapses in netiquette and their implications.

**Conclusions:**

Based on the data accrued, this article proposes basic guidelines on netiquette and measures to support their effective employment. This includes curricular adaptations, methods of teaching and enhancing engagement with the students and faculty training. Drawing on prevailing studies, it also recommends methods of assessing netiquette, online professionalism, and the learning environment. Suggestions are also made for future areas of study.

## Introduction

Observing appropriate digital social norms or netiquette has been far from simple. The online training environment has unique traits, conditions, professional standards, codes of conduct, roles and responsibilities, implicit norms, culture, values, beliefs, and principles^1–9^ (henceforth netiquette).^10^ This environment offers upcoming physicians a unique and regulated learning environment that inculcates them with the requisite knowledge, skills, and attitudes needed for the competent future physician. However, the sudden adoption of netiquette as teaching moved to online platforms to adapt to the COVID-19 pandemic has also compromised learning.^11–15^ Confounded by unclear program expectations, roles and responsibilities, as well as rules of conduct expected of users, medical students have struggled to adapt to professional, social, and clinical expectations on participation during online teaching. Of note, these struggles have also resulted in unprofessional behavior,^16^ compromised interpersonal relationships,^17^ disengagement,^18–21^ and poor learning environments.

Even now, as COVID-19 restrictions have begun to ease and as in-person teaching has resumed, online teaching continues to be employed. To address this gap and sustain online professionalism and etiquette (netiquette) to guide online communication, clinical teaching, and build a conducive online learning environment,^22–24^ a review of current netiquette guidelines is proposed.

## Methodology

We adopt Krishna's Systematic Evidence-Based Approach (SEBA) to guide a wide-ranging review of netiquette guidelines. The constructivist approach^25,26^ and relativist lens^27,28^ are best placed to account for the regnant social, cultural, ethical, legal, professional and contextual considerations,^29,30^ alongside the desired characteristics and expectations set out by professional, organizational and ethico-legal practice that shape netiquette guidelines^31^ and how netiquette guidelines are interpreted by the organization and medical students. Furthermore, compliance with netiquette guidelines is contingent upon factors such as the medical student's narratives,^32,33^ belief systems,^34^ competencies, context, experience, goals and motivations, as well as duration of their interactions within the program.^35^ These factors affirm the SEBA's ontological and epistemological roots.

**Figure 1.**
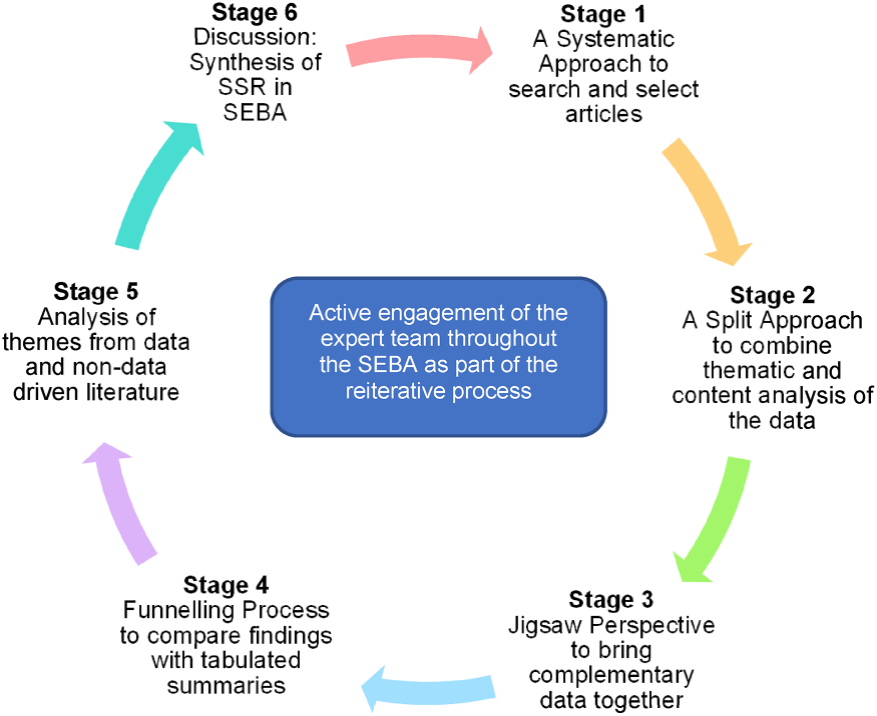
The SSR in SEBA process. Abbreviations: SEBA, Systematic Evidence-Based Approach; SSR, systematic scoping review.

A SEBA-guided systematic scoping review (SSR) (henceforth SSR in SEBA) methodically maps existing data, structures the extraction of key characteristics of netiquette, synthesizes and summarizes actionable and applicable information.^36–40^ This complex process relies on SEBA's 6-stage process which is illustrated in Figure 1.

An expert team comprising of a librarian from the National University of Singapore's (NUS) Yong Loo Lin School of Medicine (YLLSoM) and local educational experts and clinicians at YLLSoM, National Cancer Centre Singapore, Palliative Care Institute Liverpool, and Duke-NUS Medical School were recruited to provide a balanced review, ensure transparency and accountability, and oversee all aspects of the SEBA cycle.

### Stage 1 of SEBA: Systematic Approach

The review was guided by a PCC (Population, Concept, Context)^41^ framework. The PCC framework was employed to guide the primary research question, “*What is known about netiquette in medical schools?*” and the secondary research question, “*What are the features, causes and implications of lapses in netiquette in medical schools?”* (Table 1).

**Table 1.** PCC, inclusion, and exclusion criteria applied to database search.

PCC	Inclusion criteria	Exclusion criteria
**Population**	Undergraduate and postgraduate medical students within the clinical and/or medical settings	Practicing physicians Resident physicians, fellows Teaching faculty, master's programmes, higher education programmes Allied health specialities such as pharmacy, dietetics, chiropractic, midwifery, podiatry, speech therapy, occupational, and physiotherapy Non-medical specialities such as clinical and translational science, alternative and traditional medicine, veterinary, dentistry Non-medical students
**Concept**	Various standards of netiquette/etiquette/professionalism in online learning/virtual environments set out by analyzing: Standards for virtual/online meeting or tutorials netiquette/etiquette/professionalism Impact of standards used in virtual/online meeting or tutorials Infringement of standards in virtual/ online meeting or tutorials Suggestions on how to facilitate a more conducive/optimal online learning experience Assessing online professionalism and netiquette	Virtual reality, virtual simulations, web-modules without interaction between tutors and students, videos, podcasts Online patient education, web-based patient education, public education Continuing medical education, professional development Aspects of clinical research (disease, treatment, epidemiology) Global health or public health
**Context**	Virtual/online meetings or tutorials or video conferencing in the context of distance education	Face-to-face education, didactic education, hands-on teaching, on-site teaching

Abbreviation: PCC, Population, Concept, Context.

Independent searches were conducted on PubMed, SCOPUS, ERIC, Google Scholar and Embase databases between September 12, 2021 and January 1, 2022 for articles on online professionalism and standards of practice in online interactions within medical schools, published between January 1, 2000 and December 31, 2021. The review was restricted to papers published after 2000^42^ to facilitate a sustainable research process. The full search strategy may be found in *Supplemental Appendix A*.

For every 100 articles in a particular database, the medical students, peer-mentor, and senior researcher compared their findings at an online meeting. Sandelowski and Barroso’s^43^ “negotiated consensual validation” was used to achieve consensus on the final list of titles to be reviewed. The process was repeated where consensus was again required; interrater reliability was not evaluated.

### Stage 2 of SEBA: Split Approach

The “Split Approach”^25,42–44^ was employed to enhance the reliability of the data analyses. Two groups of researchers independently analyzed the included articles. The first team analyzed the included articles using Braun and Clarke^45^'s approach to thematic analysis by identifying common characteristics, creating codes to analyze the rest of the articles, and subsequently categorizing the codes into categories, and the categories into themes that best represent the data.^46^ The research team employed Sandelowski and Barroso’s^43^ “negotiated consensual validation” approach to determine a final list of themes.

The second team utilized a priori codes and categories derived from Taggar et al’s^47^ “*Clinical placements in General Practice: concepts and considerations of implementing remote virtual placements in the COVID world*” and Ahmed et al's^48^ “*Model for utilizing distance learning post-COVID-19 using (PACT)™ a cross sectional qualitative study*” to guide the concurrent use of Hsieh and Shannon's^49^ approach to directed content analysis. The aim of directed content analysis was to address the shortcomings of thematic analysis, including the omission of new considerations^36–38^ and negative findings.^50,51^

### Stage 3 of SEBA: Jigsaw Perspective

The identified themes and categories were viewed as pieces of a jigsaw puzzle. Guided by phases 4 to 6 of France et al’s^52^ adaptation of Noblit et al’s^53^ 7 phases of meta-ethnography, complementary pieces of the jigsaw were combined into themes and categories (Table 2).

**Table 2.** Themes and categories.

Themes and subthemes	Categories and subcategories
Guidelines for netiquette: - Safety guidelines; confidentiality and privacy guidelines; behavioral and technical guidelines	Guidelines for netiquette: - Location for consultation; technical requirements; privacy and confidentiality guidelines; dress code; behavioral guidelines
Contributing factors to reduced netiquette: - Infrastructural and logistical issues; curriculum issues; behavioral and psychosocial issues faced by students; nature of online learning environments	Contributing factors for reduced netiquette: - Faculty; students; social issues; curriculum; logistics
Implication of reduced netiquette: - Disrupted learning; disrupted interpersonal relationships	Implication of reduced netiquette: - Disrupted teaching; disrupted interpersonal relationships
Manifestations of compromised netiquette: - Technical lapses; behavioral lapses	

### Stage 4 of SEBA: Funneling

The Funneling Process compared the final themes/categories with the compiled summaries of the included articles to ensure that the data effectively represented current thinking. This process created domains that guided the discussion process in stage 6 of the SEBA.

### Results

**Figure 2.**
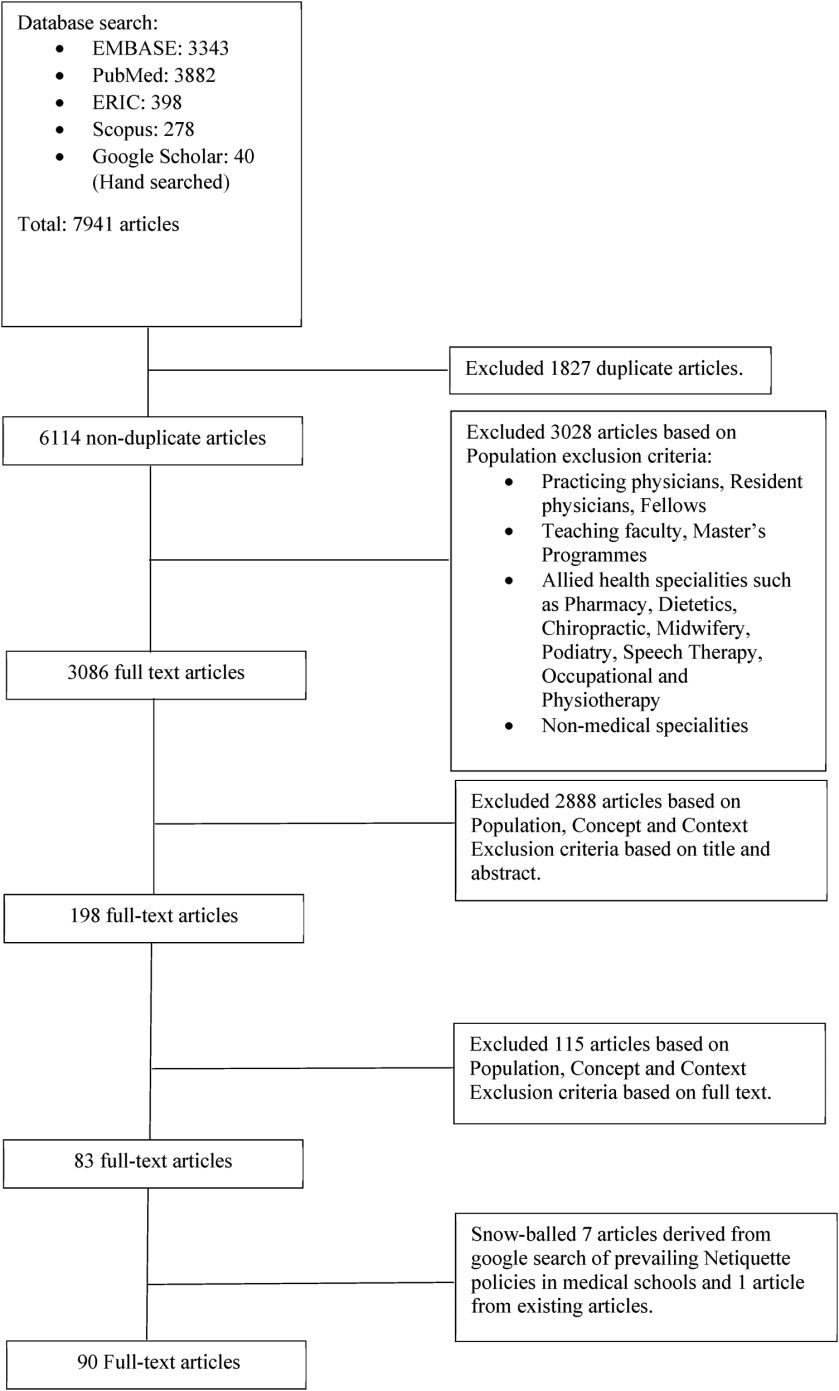
PRISMA flow chart.

In total, 7941 abstracts were reviewed, 198 full text articles were evaluated, and 83 articles were included (Figure 2). A further 7 additional articles were snowballed from existing articles to yield a total of 90 included articles. We also reviewed online guidelines of the top 10 ranked medical schools on the Times Higher Education 2022.^54^

Forty articles were quantitative studies, 14 were qualitative studies, 4 were mixed studies, 25 were descriptive/opinions/proceedings/reviews/perspectives/monographs and 7 were university guidelines. The themes and categories identified overlapped with current guidelines, contributing factors, and their implications. A thematic analysis also highlighted the manifestation of breaches in online professionalism.

## Results

The domains identified were: (1) current guidelines, (2) manifestations of poor netiquette, (3) contributing factors, and (4) implications.

### Domain 1: Current Netiquette Guidelines

A review of publicly accessible online netiquette guidelines among the top 10 ranked medical schools on the Times Higher Education 2022 list did not reveal any results.^54^ Among the 90 included articles,^17,47,48,55–62^ guidelines varied on key issues, such as confidentiality, safety, and awareness of online personas.^63–65^ These variations are credited with the rise in breaches in professionalism and standards of practice (Table 3).^24,47,66^

**Table 3.** Existing guidelines for online professionalism and netiquette.

Broad themes	Specific guidelines	Rationale	References
Safety	Institutional login or reporting attendance	Prevent false identities	61,67,68
	Keep personal passwords private; do not login with other users’ credentials	Prevent false identities	69
Awareness of online persona	Professional backgrounds for video meetings	Avoid distractions in the background	47,70
	Standardise format of names	Effective communication and identification	66
	Do not misrepresent identity	Prevent false identities	69,71
	Punctuality	General professionalism	72
	Quiet and private workspace	Avoid distractions in the background	47,66,70,72
	Stable internet connection	Avoid cutting off in the middle of conversations	70
	Keeping camera on	Active discussion and participation	64,66,72–74
	Uploading photo as a display picture	Maintains a “presence”	64,75
	Wear headphones/effective, and private audio input and output	Maintain privacy, confidentiality, effective communication and nurtures a safe physical environment	47,76
	Appropriate attire	General professionalism, changing clothes as a mechanism by which students “switch on professional identity”	47,66,72,77,78
	Mobile usage is not permitted if it compromises patient care and privacy	Diverting attention away from others, uninvolved, compromised care	47
	Asking questions should be done via the chat function if someone else is speaking	Minimize disruptions to the speaker	68,79,80
	Ask specific questions	Allows for robust conversation and reduces misunderstanding	81
	Communicate with respect, do not harass, defame, or speak in obscene language to others	Reduce disrespectful and dismissive interactions	69,71,82,83
	Do not disrupt the learning environment deliberately	Allows for a more conducive and less disruptive learning environment	71
	Communicate with respect	Reduce disrespectful and dismissive interactions	81
	Do not attach unnecessary files	Potentially sensitive material might be broadcasted, and malware may be sent	82
	Do not multitask	Being present and actively engaging in the meeting	72
Confidentiality	No recording without explicit consent from all participants	Maintains confidentiality	47
	Do not copy a message or attachment without permission	Potentially sensitive material might be broadcasted	82
	Do not disclose patient identity, patient data, or patient images	In keeping with HIPAA and other applicable laws and policies governing patient confidentiality	83,84

### Domain 2: Manifestations

The lapses in netiquette fall into 2 groups—technical and individual lapses (Table 4).

**Table 4.** Manifestations of compromised online professionalism and netiquette.

Lapses	Manifestation	References
Technical	Unstable internet connection	85–89
	Disruptive environments	85,88,90
	Non-members invading virtual meetings	81
	General technical issues e.*g., other connection, communication and visual issues that are not conducive for online learning and discussion and interrupt effective participation*	88,91
Individual	Inadequate environment for partaking in online meetings	85,88,90
	Failure to turn on/turn off videos during discussion	59,62,73
	Lack of attention/increased disengagement	92–94
	Lack of effective participation	73,93,95
	Distractibility during online lessons and discussions *e.g. multitasking*	75,92

Individual lapses in netiquette included poorly selected and inappropriate learning environments,^85,88,90^ failure to turn on their videos,^59,62,73^ poor participation,^73,93,95^ and a lack of attention during lessons and discussions.^75,92–94^

Technical lapses included an unstable internet connection^85–89^ and inadequate and/or inappropriate devices hindering students’ effective participation in online teaching.^88,91^ Disturbing instances of hackers invading virtual meetings and sharing explicit comments and images, while uncommon, were also reported.^81^

### Domain 3: Contributing Factors

Aside from a lack of consistent guidelines^23,96^ and effective assessment tools,^97,98^ breaches in netiquette include technical gaps, curriculum limitations, poor means of identifying and supporting poorly motivated students,^90,99–101^ students with mental health issues and behavioral problems,^90,101–104^ burnout,^91,99,102^ loneliness,^101,105^ cynicism,^102^ arrogance and frustration,^101^ distraction,^62,101,106–108^ lack of self-discipline,^107^ stress and anxiety (Table 5).^101,103,105,109–112^

**Table 5.** Possible contributing factors for reduced online professionalism and netiquette.

Theme	Possible factor	References
Infrastructural	Lack of adequate, robust, and accessible infrastructure	17,85,87,113–120
	Poor technical skills	88,117,121
Teaching issues	Lessons dissatisfaction due to methods of instruction (teaching style, lesson type, teaching pace)	85,87,101,105,122–125
	Lessons are too long	85,88,126
	Topics are too difficult	91,127
Time management issues	Poor scheduling/conflicts in scheduling	17,128,129
	Overall time commitment is too much	85,126
Mental health	Reduced motivation	90,99−101
	Burnout	91,99,102,130
	Stress (academic, psychosocial, familial) and anxiety	101,103,105,109–112,131
	Loneliness	101,105
	Mental health deterioration	90,101–104
Behavioral change	Cynicism	102
	Arrogance/irritation and frustration	101
	Distractions and reduced concentration	62,101,106–108
	Lack of self-discipline	107
Nature of online platforms	Inability to read nonverbal cues	88,132
	Intrusion of privacy	78
	Health issues from viewing laptops and computer	86,87
	Students’ unawareness of when to be professional	78

Technical lapses result from inadequate facilities,^91,127^ poor internet access,^85–88^ limited access to computers,^17,85,87,117–120^ restrictions in the number of private rooms and conducive online learning environments.^85,88,90^ Other sources of technical lapses include poor faculty training,^85,87,101,105,123–125^ skills management,^88,117,121^ and time limitations.^17,129^

### Domain 4: Implications

While the short-term implications have focused on disrupted and ineffective learning,^23,59,73,87,133–137^ it is perhaps equally concerning that poor netiquette is also associated with poor tutor-learner relationships,^48,62,75,94,95^ reduced peer interactions^17,95,138^ and increased student isolation.^95,105,139,140^ These concerns platform a tendency toward reduced collegiality,^76,139^ poor faculty relations^105,141^ and ineffective guidance, assessment and support of learners.

### Stage 5 of SEBA: Analysis of Evidence-Based and Non-data Driven Literature

Almost half the included articles were data-driven while the remaining articles were non-data driven. A unique aspect of the SEBA is that it allows the inclusion of articles such as position, perspective, conference, reflective and opinion papers, editorials, commentaries, letters, posters, oral presentations, forum discussions, interviews, blogs, governmental reports, policy statements and surveys. Two considerations emerged from the analysis of these articles.

First, there were concerns that the non-data-based articles may have introduced bias to the analysis. Themes drawn from the data-driven publications were thus compared with those from non-data-based articles and the similarities between the 2 groups revealed no untoward biases.

Second, there were also questions over the quality of the included articles. While MERSQI and COREQ quality appraisals were carried out, deeper consideration of the data-driven articles were called for. Some articles only discussed the notion of netiquette in passing.^12,55–57,59,61,62,70,76,79,80,85,86,90,96–98,100,101,108–110,118,120,121,124–127,134–137^

Out of the articles that touched on netiquette, 22 articles discussed netiquette and 6 articles evaluated the issue of lapses in netiquette.

## Discussion

### Stage 6 of SEBA: Synthesis of Discussion

The “Best Evidence Medical Education (BEME) Collaboration Guide”^142^ and the “Structured approach to the Reporting In healthcare education of Evidence Synthesis (STORIES)”^143^ were used to guide the discussion.

In addressing its primary research question on “*What is known about netiquette in online medical education?*” and the secondary research question, “*What are the features, causes and implications of lapses in netiquette in medical schools?”*, this SSR in SEBA highlights that the lapses in netiquette are dynamic and multifactorial. While much has been made of the absence of a uniformly accepted set of netiquette guidelines and assessment tools,^97,98^ it is perhaps more striking that most available guidelines fail to consider the impact of sociocultural considerations on online conduct and expectations.^63^ This is further compounded by a lack of due considerations on the dynamic and context-specific nature of online teaching. These latter points underline the need for netiquette guidelines to take a generalized perspective and to be infused by local, contextual, and subject considerations.

To this end, we outline a set of recommendations for addressing online professionalism in Table 6.

**Table 6.** Baseline expectations for online professionalism and netiquette.

**Security and confidentiality**
Use institutional logins when using teleconferencing platforms Change name to accurately reflect identity and the use of unique personal identifiers (matriculation ID) Turn on cameras when reporting attendance No digital recording or screen-shotting by students No partaking in tele-consults or lessons involving patient data in public areas
**Environment**
Appropriate infrastructure^58,60^ Access to adequate facilities^70,106^ Institutions must proffer evidence-based training^22,58,61,108,118,139^ to tutors^48,144,145^ and students^76^ on effective online learning^59^ Educate participants on the benefits of online training,^62,91,119,146^ engagement,^62,109^ critical reflection,^62^ and adopting and displaying professional values, beliefs, principles and concepts^147^ Emphasise and police netiquette,^23,148^ standards of privacy and confidentiality^148,149^ Provision of accessible psychological support as needed^90,101−104^ Limited to 3 online classes per day^86^ to reduce fatigue, digital eye strain^62,86^ and headaches^86^
**Awareness of online persona**
Use professional backgrounds for video meetings Taking calls at private and quiet workspaces and not on-the-go (eg, while driving, walking from point to point, in public transport) Having stable internet connection; keep cameras on throughout the discussion Uploading photos as a display picture (if video feed is not working) Wear headphones with adequate audio input and output Appropriate attire and decorum No using other devices when interacting with patients (if it compromises care, confidentiality, and privacy) Asking questions should be done at appropriate times to not disrupt the speaker Questions should be specific and directed to the appropriate audience No using chat function to communicate with other students privately Be respectful when conversing with others, do not be dismissive or disrespectful No typing or verbalizing of derogatory or offensive statements in the chat, be it privately or publicly
**Approach**
Pre-recorded webinar presentations^149^ with interspersed reflection questions and a brief postmodule quiz Holding video conferences^150^ with small groups of students to guide behaviors and rules of professionalism with other students Using simulated encounters with simulated patients^151^ Using a problem-based learning approach that allows students to re-evaluate their own digital identities and their interpretation of guidelines^152^ Observing role models as another means of influencing behavioral change^78^ Asynchronous pre-recorded lectures and short videos, as well as synchronous live-streamed lectures while low bandwidth strategies include asynchronous assignments and quizzes, alongside synchronous collaborative tasks “Warm calls” see students forewarned that they will be required to answer an upcoming question^62^; assigning students with specific roles in each session,^153^ pauses (at least 10 s) given to provide time for students to switch on their microphones to ask questions^132,133,154^; and synchronous, real-time assessments incorporated into online activities to ensure that core concepts are clearly understood^64,132,133,154^ Livestreaming in anatomy halls,^155^ using whiteboard annotation functions,^132^ role modeling^78^ and regular small group interactions^145^ will also enhance engagement^153^ and foster coordinated active participation^133,156^ Assessments may be focused in the following aspects: (1) compliance with the laws and regulations governing cyberspace (2) individual professionalism in using cyberspace (3) knowledge management and information literacy (4) respect for professionalism in interpersonal and group rules (5) complying with ethics in the use of cyberspace (6) Conduct, perceptions and attitudes toward netiquette during group discussions and presentations

## Limitations

Netiquette in medical education is a relatively under-reviewed and novel area in literature which may account for the limited papers considered in this article. Furthermore, our specific focus on the presence of netiquette guidelines available online may have precluded the inclusion of guidelines that are only made available on university intranet systems, accessible to students and faculty.

Due to time, manpower and resource constraints, the exclusion of other health professional literature may have also led to the omission of key ideas potentially transferable to the field of medical education in medical schools. This is especially important, given that there may be a common set of guidelines that involve all healthcare professionals.

Including articles in or translated into English may have also restricted search results. As most of the data was drawn from North America and the Europe, they may not necessarily be transferable beyond these regions. While we advance a general expert opinion-guided set of guidelines, more context-specific adaptations may be needed to account for local healthcare, education, and sociocultural considerations.

## Conclusion

This SSR in SEBA highlights the complex, variable, and multifactorial factors that impact current online professionalism and netiquette guidelines.

In our tabulated recommendations for Online Professionalism and Netiquette (Table 6), we have addressed not only the lack of standardized guidelines or assessment tools, but factors which are determined by sociocultural considerations, such as teaching approaches and support for students. Having said that, this framework will require further adaptation to local social and cultural settings, program nature and goals, student profiles and characteristics, as well as infrastructural considerations, for it to be effective. There is scope to pilot this framework and appraise its intervention for further improvement.

## Supplemental Material

sj-docx-1-mde-10.1177_23821205241255268 - Supplemental material for Enhancing Professionalism Online (Netiquette) in Medical Schools: A Systematic Scoping Review Supplemental material, sj-docx-1-mde-10.1177_23821205241255268 for Enhancing Professionalism Online (Netiquette) in Medical Schools: A Systematic Scoping Review by Donovan Kai Wei Ng, Jonathan Zhen Liang, Ruth Si Man Wong, Vijayprasanth Raveendran, Gillian Li Gek Phua, Warren Fong, Crystal Lim, Jamie Xuelian Zhou and Lalit Kumar Radha Krishna in Journal of Medical Education and Curricular Development

sj-docx-2-mde-10.1177_23821205241255268 - Supplemental material for Enhancing Professionalism Online (Netiquette) in Medical Schools: A Systematic Scoping Review Supplemental material, sj-docx-2-mde-10.1177_23821205241255268 for Enhancing Professionalism Online (Netiquette) in Medical Schools: A Systematic Scoping Review by Donovan Kai Wei Ng, Jonathan Zhen Liang, Ruth Si Man Wong, Vijayprasanth Raveendran, Gillian Li Gek Phua, Warren Fong, Crystal Lim, Jamie Xuelian Zhou and Lalit Kumar Radha Krishna in Journal of Medical Education and Curricular Development

sj-docx-3-mde-10.1177_23821205241255268 - Supplemental material for Enhancing Professionalism Online (Netiquette) in Medical Schools: A Systematic Scoping Review Supplemental material, sj-docx-3-mde-10.1177_23821205241255268 for Enhancing Professionalism Online (Netiquette) in Medical Schools: A Systematic Scoping Review by Donovan Kai Wei Ng, Jonathan Zhen Liang, Ruth Si Man Wong, Vijayprasanth Raveendran, Gillian Li Gek Phua, Warren Fong, Crystal Lim, Jamie Xuelian Zhou and Lalit Kumar Radha Krishna in Journal of Medical Education and Curricular Development
