# Supplementary material for: Enhancing Professionalism Online (Netiquette) in Medical Schools: A Systematic Scoping Review
Source: J Med Educ Curric Dev. 2025 Feb 24;12:23821205241255268. doi: 10.1177/23821205241255268 (PMC11851755; doi:10.1177/23821205241255268)
Supplement: sj-docx-1-mde-10.1177_23821205241255268 - Supplemental material for Enhancing Professionalism Online (Netiquette) in Medical Schools: A Systematic Scoping Review [file sj-docx-1-mde-10.1177_23821205241255268.docx]

**Full text Pubmed Search Strategy**

("education, distance"[MeSH Terms] OR "Social Networking"[MeSH Terms] OR (("online"[Title/Abstract] OR "virtual"[Title/Abstract] OR "digital"[Title/Abstract] OR "remote"[Title/Abstract] OR "virtual meeting"[Title/Abstract] OR "videoconferencing"[Title/Abstract] OR "video conferencing"[Title/Abstract]) AND ("learning"[Title/Abstract] OR "education"[Title/Abstract] OR "e-learning"[Title/Abstract] OR "zoom"[Title/Abstract] OR "classroom"[Title/Abstract]))) AND ("education, medical"[MeSH Terms] OR "students, medical"[MeSH Terms] OR (("medical"[Title/Abstract] OR "medicine"[Title/Abstract] OR "clinical"[Title/Abstract]) AND ("student*"[Title/Abstract] OR "undergraduate*"[Title/Abstract] OR "postgraduate*"[Title/Abstract]))) AND ("Interprofessional Relations"[MeSH Terms] OR "Ethics"[MeSH Terms] OR "Behavior"[MeSH Terms] OR "Professionalism"[MeSH Terms] OR "netiquette*"[Title/Abstract] OR "etiquette*"[Title/Abstract] OR "professional*"[Title/Abstract] OR "behaviour*"[Title/Abstract] OR "behavior*"[Title/Abstract]) AND ("sars cov 2"[MeSH Terms] OR "covid 19"[MeSH Terms] OR "Coronavirus Infections"[MeSH Terms] OR "2019-nCoV"[Title/Abstract] OR "2019-nCoV"[Title/Abstract] OR "nCov"[Title/Abstract] OR "covid 19"[Title/Abstract] OR "covid19"[Title/Abstract] OR "COVID-2019"[Title/Abstract] OR "COVID2019"[Title/Abstract] OR "sars cov 2"[Title/Abstract] OR "sars cov 2"[Title/Abstract] OR "sarscov2"[Title/Abstract] OR "sarscov-2"[Title/Abstract] OR "sars-coronavirus-2"[Title/Abstract] OR "sars corona virus"[Title/Abstract] OR "sars like coronavirus"[Title/Abstract] OR "novel coronavirus"[Title/Abstract] OR "novel corona virus"[Title/Abstract] OR "covid*"[Title/Abstract] OR "coronavirus-2"[Title/Abstract] OR "coronavirus infection*"[Title/Abstract] OR "coronavirus disease"[Title/Abstract] OR "corona virus disease"[Title/Abstract] OR "new coronavirus"[Title/Abstract] OR "new corona virus"[Title/Abstract] OR "new coronaviruses"[Title/Abstract] OR "novel coronaviruses"[Title/Abstract] OR "severe acute respiratory syndrome coronavirus 2"[Title/Abstract] OR (("coronavirus"[Title/Abstract] OR "nCov"[Title/Abstract] OR "sars-cov"[Title/Abstract]) AND ("2019"[Title/Abstract] OR "19"[Title/Abstract] OR "Wuhan"[Title/Abstract])))
