# Supplementary material for: Enhancing Professionalism Online (Netiquette) in Medical Schools: A Systematic Scoping Review
Source: J Med Educ Curric Dev. 2025 Feb 24;12:23821205241255268. doi: 10.1177/23821205241255268 (PMC11851755; doi:10.1177/23821205241255268)
Supplement: sj-docx-2-mde-10.1177_23821205241255268 - Supplemental material for Enhancing Professionalism Online (Netiquette) in Medical Schools: A Systematic Scoping Review [file sj-docx-2-mde-10.1177_23821205241255268.docx]

**Appendix B: Tabulated Summary Table with MERSQI and COREQ**

| **No.** | ***Author/Year*** | **Ref**  **No.** | **Article Title** | **Study Design** | **MERSQI** | **COREQ** | **Study Aim** | **Methodology** | **Key Findings** | **Proposed Solutions/ Conclusions** |
| --- | --- | --- | --- | --- | --- | --- | --- | --- | --- | --- |
| **1** | *Afonso, J. S., et al. 2018* | 125 | Pedagogical mediation using the virtual learning environment and the new generation: A search for improved performance in medical education | Quantitative and Qualitative Case Study | 10 | 12 | The purpose of this work was to describe and contextualize the application of active pedagogical methodologies using the virtual learning environment (VLE) as a tool to improve the students’ performance. | This research is a quantitative and qualitative case study, carried out in one faculty of medicine in the state of Amazonas. Given a total population of senior students in this faculty, the number of participants corresponds to a proportion of 75% in the period of two years. This work required a paradigm shift, by focusing on interactive and technological teaching, professionalism, mentoring, communication, and feedback. Courses using virtual learning environment (VLE) should be prone to interaction, dialogue, collaboration and, asynchronously, autonomy. One of the key aspects of VLE content production is to select appropriate subject matters and texts to be discussed. It is through the VLE contents that the mediation of knowledge construction process and the participants’ interaction takes place. | The improvement over the pre-test evaluation was evident, ranging between 12.59 and 50.9%, depending on the subject matter, to 81.31% in the final evaluation. In addition, the students expressed consistently positive opinions regarding the use of VLE | The use of Virtual Learning Environments as a pedagogical tool, in an interactive format and appropriate to the transmission of information and knowledge construction, was adequate for an evident improvement in the cognitive performance of the senior students of medicine. In addition, the positive evaluation of the students regarding the pedagogical methodology used constitutes an added value of this approach. |
| **2** | *Agnelli, B., et al.2022* | 114 | Blended practical learning in compliance with COVID-19 social distancing | Qualitative | NA | 24 | The purpose of the to report the conversion of an Educational Model, into an effective blended learning format for the post-pandemic era. Satisfaction with this learning model by students and tutors was then evaluated with a survey. | The study provided two ad hoc satisfaction questionnaires, one for students, and one for tutors, with both open and closed questions. The closed questions are based on a Likert scale, from 1 to 10 (1=not satisfied at all, 10=extremely satisfied), including “not applicable” (N/A), to quantitatively evaluate the impact of this project. The open questions, on the other hand, leave an empty space where students and tutors could leave a comment to perform a qualitative analysis, obtaining further sug- gestions or criticisms. | The interaction between teachers and students, as well as among students themselves, was rated more than sufficient, 7.1 and 6.2, respectively. The strongest point, which was evaluated with the highest score, is the availability of tutors to discuss the provided material, with a 8.2. The qualitative analysis of data, a sample of which is presented in Fig. 6, derived  from comments and suggestions proposed by our undergraduates and faculty, confirms the advantage of the blended learning activities in order to guarantee a continuation of the clinical curriculum, although it highlighted the necessity for an increased technical support for connection problems and an improvement in organization of the meetings | Concerning the evaluation questionnaire, students, as well as tutors, identified, in all courses, a good balance between online and face-to-face meetings and recognized the method to be helpful to reach the learning objectives. We believe that this blended method is beneficial for students rather than a purely online learning, especially concerning practical activities. This is in line with what educationalists already understand about the impact of blended learning |
| **3** | *Ahmed, S. A., et al. 2020* | 49 | Model for utilizing distance learning post COVID-19 using (PACT)™ a cross sectional qualitative study | Qualitative | NA | 16 | This work aims at mapping priorities for post-COVID planning for better balance between distance learning and face to face learning. | A virtual mapping session was held with 79 faculty from 19 countries. They worked in small groups to determine upsides and downsides of face-to-face and DL subsequently. An initial polarity map was generated identifying five tension areas; Faculty, Students, Curriculum, Social aspects and Logistics. A 63-item assessment tool was generated based on this map, piloted and then distributed as a self-administered assessment. The outcomes of this assessment were utilized for another mapping session to discuss warning signs and action steps to maintain upsides and avoid downsides of each pole. | Participants agreed that face-to-face teaching allows them to inspire students and have meaningful connections with them. They also agreed that DL provides a good environment for most students. However, students with financial challenges and special needs may not have equal opportunities to access technology. As regards social issues, participants agreed that face-to-face learning provides a better chance for professionalism through enhanced team-work. Cognitive, communication and clinical skills are best achieved in face-to-face. Participants agreed that logistics for conducting DL are much more complicated when compared to face-to-face learning. Participants identified around 10 warning signs for each method that need to be continuously monitored in order to minimize the drawbacks of over focusing on one pole at the expense of the other. Action steps were determined to ensure optimized use of in either method. | In order to plan for the future, we need to understand the dynamics of education within the context of polarities. Educators need to understand that the choice of DL, although was imposed as a no-alternative solution during the COVID era, yet it has always existed as a possible alternative and will continue to exist after this era. The value of polarity mapping and leveraging allows us to maximize the benefit of each method and guide educators’ decisions to minimize the downsides for the good of the learning process. |
| **4** | *Ahmed, S. A., et al. 2020* | 120 | A Step-by-Step Guide to Managing the Educational Crisis: Lessons Learned from COVID-19 Pandemic | Opinion | NA | NA | This article aims to provide tips that can provide a guide for medical education leaders to coordinate crisis management referring to the Egyptian context. | This work was done using a reflection on the COVID-19 response by Egyptian universities and analysis of such responses. | Medical Education Institutions are required to build a taskforce team for crisis management. These should be committed to supporting sudden online education transition, academic support, and the psychological well-being of students, staff members, health care professionals, paramedics, and faculty administration. As the situation evolves, the taskforce has to monitor the challenges and provide appropriate plans, guidance, and solutions. Leaders in medical education have a crucial role in response to the pandemic crisis in securing a successful educational process while ensuring the mental and psychological well-being of the stakeholders | Crisis management is the skill of the future and more investment needs to be placed in designing crisis response and in enabling universities to. Managing higher education institutions regarding crisis require a new set of skills. There are twelve areas to focus during crisis. It is recommended to use a daily checklist to follow-up the work stream |
| **5** | *AlQhtani, A., et al. 2021* | 139 | Online versus classroom teaching for medical students during COVID-19: measuring effectiveness and satisfaction | Quantitative | 10 | NA | Aimed to measure the effectiveness of e-learning during the COVID-19 pandemic, as well as medical students’ preferences regarding e-learning and classroom teaching, and the possibility of applying it post-pandemic. | A cross-sectional online survey of medical students (N = 376) in six medical schools was carried out after their second semester, from August 15 to 20, 2020. Ten parameters were measured for the effectiveness of eLearning based on a 5-point Likert-scale and five parameters were measured for satisfaction. | E-learning was more or equally effective in four parameters such as assignment submission and meeting individual needs, but less effective in six parameters, including building skills and knowledge, and interaction level. Satisfaction was either high or neutral in all five parameters. | Findings have shown that e-learning can assist the teaching process in medical schools in some respects, but cannot be used for the entire teaching process. |
| **6** | *Ashokka, B., et al. 2020* | 68 | Coordinated responses of academic medical centres to pandemics: Sustaining medical education during COVID-19 | Descriptive | NA | NA | State the consolidated and systematic approach for academic medical centres in response to the evolving pandemic outbreaks for sustaining medical education | - | Academic medical centres need to establish a ‘COVID-19 response team’ in order to make time-sensitive decisions while managing pandemic threats. Major themes of medical education management include leveraging on remote or decentralised modes of medical education delivery, maintaining the integrity of formative and summative assessments while restructuring patient-contact components, and developing action plans for maintenance of essential activities based on pandemic risk alert levels. These core principles must be applied seamlessly across the various fraternities of academic centres: undergraduate education, residency training, continuous professional development and research. Key decisions from the pandemic response teams that help to minimise major disruptions in medical education and to control disease transmissions include: minimising inter-cluster cross contaminations and plans for segregation within and among cohorts; reshuffling academic calendars; postponing or restructuring assessments. | While minimising the transmission of the pandemic outbreak within the healthcare establishments is paramount, medical education and research activities cannot come to a standstill each time there is a threat of one. |
| **7** | *Bachir, B., et al. 2021* | 113 | The educational and psychological impact of the COVID-19 pandemic on medical students: A descriptive survey at the American University of Beirut | Quantitative | 8 | NA | This study aimed at describing the response of medical students at AUBFM to the COVID-19 pandemic in terms of stress. | Sent all medical students at the American University of Beirut Faculty of Medicine (AUBFM) an online anonymous survey by email. The survey started with general questions (age, gender and medical school year), followed by 3 sections that contain questions pertaining to the attitudes of medical students towards clinical rotations and online classes. Data was then analysed using SPSSv24 and was then reported as percentages | The majority of clinical students (Med 3 and Med 4) reported that they feel nervous during their rotations in the hospital. Moreover, they reported that they have increased their use of disinfectants and personal protective equipment since the emergence of the pandemic. Moreover, the majority of medical students reported that they feel more stressed after shifting to online classes. Medical students also reported that they would be willing to go back to on-campus classes | Limited data exists in the literature concerning the psychological impact of the COVID-19 pandemic on medical students in the middle East. Medical students reported that they feel more stressed and nervous during their clinical rotations and after the shift to online education, affecting their academic and social life. Further studies using a larger sample size are needed. |
| **8** | *Bhattarai, B., et al. 2021* | 109 | Perception of online lectures among students of a medical college in Kathmandu: A descriptive cross-sectional study. | Quantitative | 7 | NA | The objective was to know the perception towards online lectures by the undergraduate students of a medical college. | Conducted among the undergraduate students of dentistry, medicine, and nursing at Kathmandu Medical College via self-administered online questionnaire. Data were collected from November to December 2020 after ethical clearance from institutional review committee (Ref. no. 0311202002). Students who had not attended even one hour of online learning per week were excluded. Responses were collected using Google Forms which were analysed in Microsoft Excel. Descriptive statistics are presented as means, standard deviations, frequencies, and percentages | Out of 318 valid questionnaires, 143 (44.97%) students agreed that online lectures were effective but 138 (43.4%) disagreed that online lectures were more useful than traditional lectures. One hundred and forty-five (45.60%) found online classes difficult to concentrate and 175 (55.03%) agreed that they preferred a combination of traditional teaching and online tutorials. Only two (0.63%) students strongly agreed on excellent internet during classes and 104 (32.70%) agreed it caused economic burden. Mean age of participants was 20.75±1.538 years; 202 (63.52%) were females; online learning per week was 18.75±9.157 hours; and duration of online learning was 20.28±9.997 weeks. | Most of the students had a positive attitude towards e-learning when compared to similar studies. Further multicentric studies with larger sample size would better demonstrate whether online education partly or fully can be effective adjunct to traditional face to face interaction. |
| **9** | *Brenner, K., et al. 2021* | 110 | Undergraduate and Graduate STEM Students' Experiences during COVID-19: Proceedings of a Virtual Workshop Series | Proceedings | NA | NA | Institutions of higher learning took various approaches to the COVID-19 pandemic, which raised two questions: what factors informed decisions at these institutions, and what new initiatives or practices seem to be effective for students during the COVID-19 pandemic? | This Proceedings of a Virtual Workshop Series has been prepared by Joe Alper as a factual summary of what occurred at the virtual meetings | the National Academies of Sciences, Engineering, and Medicine held a virtual workshop consisting of four online sessions that took place between September 22 and October 6, 2020. Organized by the Board on Science Education and the Board on Higher Education and Workforce, the virtual workshops provided an o | - |
| **10** | *Buelens, H., et al 2007* | 24 | Electronic discussion forums in medical ethics education: the impact of didactic guidelines and netiquette | Randomised Controlled Trial | 15.5 | NA | This paper investigates whether the type of guideline provided has an effect on either or both the quality of asynchronous group discussion and the appraisal of participants in the context of a course in medical ethics. | A total of 112 students graduating in the biomedical sciences were randomly assigned to electronic discussion groups of 18–19 participants each. Three treatment groups were created by varying the guidelines presented to participants upon entering the discussion forum. Guidelines contained trivial information, netiquette advice, or a combination of didactic guidelines and netiquette advice. | Both the quality of discussion and the participants’ appraisal of the discussion were highest in the third group. However, contrary to what is widely suggested, it was observed that exclusively supplying netiquette guidelines had no impact on discussion quality. | To improve the quality of asynchronous group discussion, it is advisable to provide not only netiquette rules, but also didactic guidelines. Future research should focus on the effects of netiquette guidelines on students of different levels and skills and should allow for disentangling the effects of netiquette and didactic guidelines. It might also seek to establish methods of disseminating guidelines that enhance their effect. |
| **11** | *Caton, J. B., et al. 2021* | 81 | Student engagement in the online classroom: comparing preclinical medical student question-asking behaviors in a videoconference versus in-person learning environment. | Quantitative Perspective | 11.5 | NA | The aim of this study is to assess whether there were differences in learner engagement, as evidenced by student question-asking behaviors between in-person and videoconferenced sessions in one preclinical medical student course | In Spring, 2020, large-group didactic sessions in POM were converted to video-conference sessions. During these sessions, student microphones were muted, and video capabilities were turned off. Students submitted typed questions via a Q&A box, which was monitored by a senior student teaching assistant. We compared student question asking behavior in recorded video-conference course sessions from POM in Spring, 2020 to matched, recorded, in-person sessions from the same course in Spring, 2019. | Found that, on average, the instructors answered a greater number of student questions and spent a greater percentage of time on Q&A in the online sessions compared with the in-person sessions. Also found that students asked a greater number of higher complexity questions in the online version of the course compared with the in-person course | The video-conference learning environment can promote higher student engagement when compared with the in-person learning environment, as measured by student question-asking behavior. Developing an understanding of the specific elements of the online learning environment that foster student engagement has important implications for instructional design in both the online and in-person setting. |
| **12** | *Cheng, X., et al. 2021* | 122 | Gross Anatomy Education in China during the Covid-19 Pandemic: A National Survey | Quantitative | 10.5 | NA | To evaluate the state of gross anatomy education in China during the pandemic, a nationwide survey was conducted through convenience sampling by email or respondent invitations on social media | A total of 359 questionnaires were received from the respondents. The first response from a given school was included in the study to represent that school, thus, 77 questionnaires were used for analyses. Schools represented were from all provinces in mainland China as well as Hong Kong and Macao. | The survey found that before the pandemic, 74.0% and 33.8% of the 77 schools conducted online theoretical and practical sessions, respectively, on gross anatomy, and 36 (46.8% of 77) had temporarily suspended practical sessions at the time the survey was conducted. Body donation programs were also affected with 26.0% and 27.3% of the 77 schools having suspended donation programs or saw a decreased number of donations. During the pandemic, 40.3% of the 77 schools kept or initiated the implementation of active learning, and online assessment was continued in 49.4% of the 77 medical schools. Another 26 (33.8%) schools initiated online assessment during the pandemic. A total of 359 answers were included for the analysis of the “teachers’ perception of the online teaching experience.” Over half (51.0%) of the 359 responded teachers were very satisfied or satisfied with the effectiveness of online teaching during the pandemic. A total of 36.2% of these respondents preferred to implement online teaching of theoretical sessions after the pandemic, and 89 (24.8%) teachers were keen to return to traditional face-to-face anatomy education. | Forward anatomy educators in China should start strategizing about how to overcome these obstacles because the duration of the pandemic moving forward is unknown. |
| **13** | *Cobbold, C. & L. Wright 2021* | 80 | Reflections on practice: A virally-infected curriculum must adapt or suffer! | Perspective | NA | NA | In this report, the authors share some evolutions in preclinical teaching of the medical sciences that have occurred in response to the SARSCoV-2 pandemic, many of which will be relevant to other degree programs. | - | Although the learning maps have been universally well accepted, early evaluation of the COVID-induced workshops was intriguing. The majority of students at the larger site indicated that they would be happy for workshops to continue online post-COVID, however more students at the smaller site indicated a preference for the pre-COVID face-to-face arrangement, when possible, instead of online instruction. In addition, almost all students at the larger site, but only a quarter at the smaller site, said they were happy for sessions to continue to be delivered to the whole cohort rather than to smaller groups multiple times. | A continual cycle of feedback and appropriate change must occur, when required, to ensure effective and efficient teaching and learning, taking into consideration equity across diverse sites and different learning attitudes. This will necessitate some element of academic risk taking. |
| **14** | *Darici, D. ,et al. 2022* | 132 | Fun Slipping into the Doctor's Role"--The Relationship between Sonoanatomy Teaching and Professional Identity Formation before and during the COVID-19 Pandemic | Qualitative and Quantitative | 10 | 24 | This study investigated two questions. First: How do on-site sonoanatomy environments affect undergraduates' development of their professional identities? It was hypothesized that variables associated with PIF would be highly relevant to on-site sonoanat- omy teaching. Second: Which impact does the shift from on-site to online-only sonoanatomy have an students' PIF? This study hy- pothesized that the range of PIF-related variables, and academic performances would decline in the online-only format. | Several sources have been used in this mixed-method study: quan- titative-, and qualitative student evaluations of the anatomy and im- aging courses and the sonoanatomy modules respectively, and the national board examination results in anatomy. The evaluation data has been continuously collected during a time-span of eight weeks after the end of each course, and each semester by means of evalu- ation and examination tools. | Students emphasized content-unrelated variables during the on-site course, such as having social interactions, performing an authentic hands-on examination and professional identity-related values. At the same time, online-only cohorts reported dissatisfaction and high stress levels. Content-unrelated variables contributed to the dissat- isfaction in the online-only format. | Missing hands-on experience in sonoanatomy did not affect the instant performance but leads to a long-term decline of pattern recognition competencies. Together, these observations caution against the uncritical substitution of hands-on courses into online-only formats, as with prerecorded videos, simula- tions or e-learning environments |
| **15** | *Duke, P., et al. 2015* | 56 | Preserving third year medical students' empathy and enhancing self-reflection using small group "virtual hangout" technology | Quantitative | 11 | NA | Conducted this study to analyze the effects of a professionalism course on third year medical students’ empathy and self-reflection (two elements of professionalism) and their perceptions about the course | Initiated a faculty-facilitated, peer small group course for third year students, creating virtual classrooms using social networking and online learning management system technologies. Students completed the Groningen Reflection Ability Scale (GRAS) and the Jefferson Scale of Empathy (JSE) before and after the course and provided anonymous online feedback. | The results of the JSE before and after the course demonstrated preservation of empathy rather than its decline. In addition, there was a statistically significant increase in GRAS scores (p50.001), suggesting that the sharing of personal narratives may foster reflective ability and reflective practice among third year students. | This study supports previous findings showing that students benefit from peer groups and discussion in a safe environment, which may include the use of a virtual group video platform |
| **16** | *Dutta, S., et al. 2021* | 88 | The Satisfaction Level of Undergraduate Medical and Nursing Students Regarding Distant Preclinical and Clinical Teaching Amidst COVID-19 Across India | Quantitative Cross-sectional, observational questionnaire-based study | 12 | NA | This study was designed with an aim to assess the satisfaction level of undergraduate medical and nursing students and analyze the associated problems faced by the students during online teaching | The questionnaire had 25 items of which 23 were questions with responses on the Likert scale and two items on views and suggestions were open-ended. The online questionnaire was shared through various messaging/mailing platforms. Overall satisfaction was assessed, and a satisfaction index was calculated for each item. Data are presented in frequencies and percentages, and SPSS was used to analyze the data | A total of 1068 students participated in the study. The majority were from the age group 21–23 years (54%) and there was almost the same number of participants from both genders. The majority of the students were medical undergraduates (n=919), were in their second year (n=669), belonged to a government institution (n=897) and used a mobile phone for their online classes (n = 871). The majority of the students were dissatisfied (42%) with no significant difference between medical and nursing students (p = 0.192). First-year students were significantly dissatisfied compared with other senior students (p = 0.005). The maximum satisfaction index (78.23%) was observed with faculties being supportive and responsive in resolving the queries and the minimum (46.39%) was observed with issues related to communication and discussion with peer students. There were 662 responses as views which mostly contained negative comments regarding interaction and focus, practical learning, teaching content, and technological/infrastructural flaws. There was major dissatisfaction regarding the practical and clinical learning. | Online learning is essential at current times but is not an effective alternative for medical and nursing education. Face-to-face classes and practical sessions along with online learning can be a viable option. |
| **17** | *Elshami, W., et al. 2021* | 115 | Satisfaction with online learning in the new normal: perspective of students and faculty at medical and health sciences colleges | Quantitative | 11.5 | NA | This study aimed to identify factors affecting student and faculty satisfaction with online learning during the COVID-19 pandemic | An online questionnaire, along with the study information sheet and consent form, was sent to the expected participants in the Medical and Health Sciences Colleges. The questionnaire was organised into two parts – socio-demographic information and satisfaction with online learning – using different satisfaction scales. It consisted of 28 items, categorised into the following subscales: student, instructor and institution. The questionnaire used a four-point Likert scale, ranging from 1 (strongly disagree) to 4 (strongly agree). | Of the students, 68.7% (n = 246) were less satisfied with online learning, and 41.6% (n = 149) would not recommend the online learning experience to others. Nevertheless, students were satisfied with the com- munication during online learning (60.9%, n = 218), and almost half the students (47.5%, n = 170) were satisfied with the flexibility afforded during online learning. | the study recommends a combination of synchronous and asynchronous online approaches, incorporating different applica- tions with the learning management systems used to engage students in online learning. Constructive and timely feedback on student performance is essential to enhancing their satisfaction with online learning. Training of faculty and orientation of students in addition to IT support will improve the satisfaction of both students and faculty. Finally, institutional support, including organisational policy, incentives and faculty development, will enhance faculty satis- faction with online teaching. |
| **18** | *Finn, G., et al. 2010* | 79 | You're judged all the time!' Students' views on professionalism: a multicentre study | Qualitative | NA | 24 | This study describes how medical students perceive professionalism and the context in which it is relevant to them | Seventy-two undergraduate students from two UK medical schools participated in 13 semi-structured focus groups. Focus groups, carried out until thematic saturation occurred, were recorded and transcribed verbatim. Data were analysed and coded using NVivo 8, using a grounded theory approach with constant comparison. | From the analysis, seven themes regarding professionalism emerged: the context of professionalism; role-modelling; scrutiny of behaviour; professional identity; ‘switching on’ professionalism; leniency (for students with regard to professional standards), and sacrifice (of freedom as an individual). Students regarded professionalism as being relevant in three contexts: the clinical, the university and the virtual. Students called for leniency during their undergraduate course, opposing the guidance from Good Medical Practice. Unique findings were the impact of clothing and the online social networking site Facebook on professional behaviour and identity. Changing clothing was described as a mechanism by which students ‘switch on’ their professional identity. Students perceived society to be struggling with the distinction between doctors as individuals and professionals. This extended to the students’ online identities on Facebook. Institutions’ expectations of high standards of professionalism were associated with a feeling of sacrifice by students caused by the perception of constantly ‘being watched’; this perception was coupled with resentment of this intrusion. Students described the significant impact that role-modelling had on their professional attitudes. | This research offers valuable insight into how Phase 1 medical students construct their personal and professional identities in both the offline and online environments. Acknowledging these learning mechanisms will enhance the development of a genuinely student-focused professionalism curriculum. |
| **19** | *Forycka, J., et al, 2022* | 131 | Polish medical students facing the pandemic —Assessment of resilience, well-being and burnout in the COVID-19 era | Quantitative | 13 | NA | Our study assesses resilience, well-being, and burnout among medical students in the COVID-19 era due to the new challenges brought by the pandemic. Several compounding fac- tors like mental health problems, use of stimulants, medical education in the COVID-19 era, the level of mistrust towards healthcare professionals, the presence of SARS-CoV-2 infections in respondents, their families, and friends, and volunteer or paid work in COVID-19 units were taken into account. | The online survey consisting of validated questionnaires assessing resilience (Resilience Scale 14; RS-14), well-being (Medical Student Well-Being Index) and burnout (Maslach Burnout Inventory) as well as self-created survey concerning mental health problems, use of stimulants, SARS-CoV-2 infection, work in COVID-19 units, medical education and social attitude towards health care professionals in the pandemic era was distributed via Facebook and other online students’ platforms. 1858 MSs from all polish medical schools agreed to fill in the survey. The study survey consisted of three validated questionnaires—Resilience Scale-14, Medical Students Well-Being Index, and Maslach Burnout Inventory—General Survey for Students, as well as self-created questions. All validated questionnaires were used in accordance with the license agreements. | The most commonly applied method of e-learning was live classes via Internet communica- tors, as reported by 956 participants (80.6%), other e-learning methods comprised recorded lectures and/or seminars, presentations shared with students, and exercises for their own work. The two main methods of e-learning that were preferred most by respondents were recorded lectures and/or seminars (preferred by 40.3% of students) and live classes via Internet communicators (preferred by 39.2% of students). Practical (i.e., laboratory classes, simulations, practical classes in anatomy) and clinical classes (classes in in-patient or out-patient settings) in the first semester of the academic year 2020/2021 were completed on a limited basis.  The vast majority of students expressed concerns over their practical skills (84.3%) and the level of theoretical knowledge (66.6%) after some months of mostly online teaching. 59.9% are worried about their performance at the Final Medical Exam—passing this exam is compulsory in order to receive a medical license, also the result of the exam is the application criterion for residency.  Students’ attitudes to online learning, motivation to learn, and behavior patterns related to novel situations in medical education were also assessed. 806 (68%) respondents confirmed that they delay their tasks and postpone their duties more often in the online teaching era. Reduced motivation to learn was reported by 934 (78.8%) participants. 596 respondents stated that they learn less than before the pandemic, 338—comparably, and 250 learn more. The level of solitude of students was assessed on a 5-point Likert scale with 1 point reflecting no solitude at all and 5 points reflecting overwhelming solitude; the median was 4 (IQR 3). | The strict limitations on students’ clinical practice and work placements imposed due to the pandemic caused decreased motivation and concerns of inappropriate lev- els of knowledge as well as questioning their abilities to pass final examinations and work as doctors [21], something that was also reported in our study. Almost two-thirds of the respon- dents presented low levels of resilience, indicating a problem with their ability to maintain or regain mental health while experiencing adversity [22] such as a global pandemic. Low resil- ience was correlated with more severe burnout, poor well-being, reduced motivation, and higher usage of stimulants during the COVID-19 pandemic. This may support the conclusion that building resilience is one of the components of creating driven, invested, and content healthcare professionals. |
| **20** | *Giordano, L., et al. 2021* | 105 | Impact of Covid-19 on undergraduate and residency training | Review | NA | NA | The purpose of the review is to evaluate how the various institutes have faced the covid-19 emergency and guaranteed the perpetuation of the learning process of resident and students. | A comprehensive search of the medical literature in PubMed and Google Scholar was performed including all the works explaining how the institutes have reorganized teaching for resident and undergraduate students. | The use of internet for the dissemination of teaching material and educational meetings has built bridges, albeit virtual, between resident and teachers. New techniques for teaching and conducting exams have been introduced. The rotating team system allowed the continuation of the teaching activity in safety. | Thanks to remodulation of the teach modalities, the massive use of internet platforms, a wise distribution of work shifts, and others, universities and hospitals have not only reduced the impact on the learning process of resident and students but also turn this pandemic into a moment of personal and professional growth for the new generation of healthcare professionals. |
| **21** | *Ghosh, S.K., et al. 2022* | 78 | Lacunae regarding dearth of dissection‐based teaching during COVID‐19 pandemic: how to cope with it? | Scoping Review | NA | 10 | A scoping review was done to identify issues related to reduced time spent in anatomy teaching sessions due to the COVID-19 pandemic, and to identify solutions to mitigate this problem | The study was conducted in the Department of Anatomy at All India Institute of Medical Sciences, Patna, India. An extensive literature search was undertaken for this study from 6 indexed databases | To overcome the deficiencies in terms of lack of dissection- based teaching amidst COVID-19 pandemic, a few measures may be adopted as interim arrangement till the time the situation improves (Fig. 1).  1. Live online streaming of real time dissection sessions from the dissection room. It is critical to ensure that these sessions simulate the physical dissection as much as possible. This can be achieved by motivating the students to maintain the good practices (associated with a physical dissection class) during online sessions also. Hence students should attend these sessions in dissection room attire (lab coat/apron/any other approved) and should not record any videos neither they should share the links/ sessions with any outsider (family/friend/close relative). They should not resort to eating or drinking while the session is going on. Moreover, they should not make any comments which is unbecoming of the ses- sion. Implementation of this measure requires a stable internet connection at both ends, which at times can be a hindrance. Online streaming of virtual dissection is also an option in institutions where physical dissection is not practised. This statement is based on the outcome analysis of virtual dissection in anatomy education…… | To overcome the deficit in terms of learning outcomes due to dearth of dissection sessions (as outlined in this study and identified as lacunae pertaining to online anatomy teaching), few interim remedial measures (coping mechanism to plug the lacunae) as detailed in this paper may be incorporated into online anatomy education programs during COVID-19 pandemic. |
| **22** | *Grand, D., et al. 2021* | 74 | Medical Student Experience and Outcomes, as Well as Preceptor Experience, with Rapid Conversion of a Preclinical Medical School Course to a Remote-Based Learning Format in the Setting of the COVID-19 Pandemic | Quantitative and Qualitative | 9 | 14 | To assess student outcomes and experiences, as well as preceptor experiences, after emergently converting a preclinical medical school renal course to a remote setting during the COVID-19 pandemic. | First-year medical student examination scores and responses to Likert-scale questions on end-of-course evaluations from the 2018–2019 (traditional) and 2019–2020 (remote) academic years were compared. Free-text responses from students and preceptors were analyzed using a qualitative summative approach to extract major themes in perceptions of remote learning. | Mean student scores on course examinations did not significantly differ between the traditional and remote settings (p = 0.23 and 0.84 respectively). Quantitative analysis of student evaluations revealed no significant difference across all items in mean Likert-scale responses. Student and preceptor free-text responses identified course leader engagement and responsiveness as essential to the success of remote-based learning. Optimal group size and online etiquette are areas that require attention. | Despite rapid conversion of a preclinical medical school renal course to a remote-based format in the setting of the COVID-19 pandemic, student scores and evaluations remain positive and largely unchanged. |
| **23** | *Haras C, Calhoun A, Olson AP, Rosenberg M. 2021* | 65 | Mindful Medical Education Online | Monograph | NA | NA | The purpose of this article is to provide recommendations  for educators to optimize their approach to online curricular transformation | N/A | Recommendations for Mindful Online  Learning:   - Establish a Framework for Effective Online Learning - Build Teaching Presence into the Online Environment - Build Social Presence into the Online Environment - Build Cognitive Presence into the Online Environment - Use Engagement Triggers that Bring Learners to the “Now” of Learning - Establish Routines that Build Practice - Use Both Low and High Bandwidth Strategies - Ensure “Threshold Concepts” are Transparent - Teach Students to Ask Good Questions - Align Measurable Learning Objectives - with Instructional Materials, Learning Activities, - Assessment, and Technology Tools - Use Low‑Stakes Assessments as Formative Practice to Enhance Memory - Embody Continuous Improvement | This paper makes a practical contribution to medical  education by identifying possible improvements to original  course design via renewed and careful attention to online  course structure, design, activities, and delivery. Almost all  the techniques identified are helpful to structuring an online  course such as frequent low-stakes assessment, threshold  concepts, transparency, item analysis, collaboration, asking better questions, can be employed in a face-to-face  classroom. |
| **24** | *Haucke, E., et al. 2020* | 90 | Application of telepresence systems in teaching - transfer of an interprofessional teaching module on digital aided communication into the block training "internal medicine" during the Covid-19 pandemic | Descriptive with Quantitative Evaluation | 6 | NA | The short article outlines the interprofessional teaching module including first evaluation results and describes the adapted block training. | In the “Internal Medicine” block training, students in a lecture hall navigated a telepresence system, which was accompanied by a physician across the ward and conducted an anamnesis via video and audio transmission without actual patient contact. | Students, physicians, and patients were open-minded about this form of communication during the Covid-19 pandemic and quickly got accustomed to the use of the telepresence system. To be able to react to technical challenges (e.g. unstable connection between the communication partners), a careful preparation of the lecturers is necessary. | In using a telepresence system, patient-oriented teaching of students in the block training “Internal Medicine” can be ensured with low-threshold technical effort during the Covid-19 pandemic. The telepresence system allows for the involvement of patients into teaching while adhering to the necessary hygiene measures. Despite technical challenges, the teaching format based on telepresence is suitable as an alternative to face-to-face teaching if actual patient contact is not possible. |
| **25** | *Hayat, A. A., et al. 2021* | 17 | Challenges and opportunities from the COVID-19 pandemic in medical education: a qualitative study | Qualitative | NA | 20 | This study aims to explain the challenges and opportunities of the COVID-19 pandemic for medical education | This qualitative study used conventional content analysis to collect data from face-to-face and semi structured interviews. The interviews continued until data saturation was reached. The participants were 12 students and 14 faculty members at Shiraz University of Medical Sciences. To ensure data rigor, we used member checks, peer checks and an external observer. | Three main categories and 15 subcategories were extracted. The findings showed that four subcategories, e.g. perception on feasibility of e-learning, standardizing of e-learning, dedicated teaching, and networking and interdisciplinary collaborations, affected the development of medical e-learning. The main opportunities from the COVID-19 pandemic for medical education were classified into five subcategories: attitudes to e-learning and adaptability, preventing students’ separation from the educational environment, documentation and monitoring education, take control of own learning, and increasing perceived usefulness. The main challenges were divided into four subcategories, e.g. noncompliance with virtual classroom etiquette, inadequate interactions, time limitations, and infrastructure defects and problems. Finally, participants believed that methods of evaluation in eLearning were more suitable for diagnosis and formative evaluations. Generally, two subcategories were extracted, e.g. formative and summative. | Medical schools have necessarily moved towards e-learning to compensate for the interruption in classroom education, such that traditional classes have been replaced with e-learning. These rapid, extensive changes in teaching and learning approaches have consequences for medical schools. |
| **26** | *Ho, J., et al. 2021* | 57 | Developing the eMedical Student (eMS)-A Pilot Project Integrating Medical Students into the Tele-ICU during the COVID-19 Pandemic and beyond | Quantitative | 11.5 | NA | In this report, the authors describe the development and initial outcomes of a teleICU elective course for post-clerkship medical students that emerged from a collaboration between students and faculty. | Both students and preceptors were asked to evaluate the rotation using anonymous REDCap-based surveys containing both multiple choice seven-point Likert scale questions and open-ended questions (Appendices B and C). In addition, students completed both pre-rotation and post-rotation electronic assessments designed by the faculty to assess medical knowledge in the critical care setting. | Knowledge of critical care and telehealth was demonstrated through improvement between pre-test and post-test scores. Professional development was demonstrated through post-course preceptor and learner feedback. | This tele-ICU rotation allowed students to gain telemedicine exposure and participate in the care of COVID patients in a safe environment. |
| **27** | *Hussain, S., et al. 2021* | 58 | Pharmacy and medical students' attitudes and perspectives on social media usage and e-professionalism in United Arab Emirates. | Quantitative Cross-Sectional | 9.5 | NA | The study objectives were to: 1) Determine the social media usage levels among medical and pharmacy students in the United Arab Emirates (UAE); 2) Characterize the students' views and perceptions, including their awareness of e-professionalism; and 3) Compare the responses in behavior between the two groups. | A cross-sectional study was performed on 575 undergraduate students from two study disciplines, pharmacy (n = 325) and medicine (n = 250). Minor revisions were made to previously validated assessment tools and pilot tested. The study sample included students from five different universities across the country. | In comparison to medical students, pharmacy students were observed to use social media more for learning purposes (x2 = 6.8, p < .05). However, medical students' opinions reflected more strongly on the context of accountability and e-professionalism (x2 = 15.8, p < .05). A considerable proportion (89%) of students felt it was discriminatory for prospective employers to use their social media profile information for investigative purposes while hiring. One-third of respondents reported sharing information that they would not want their employers to view, and 67.1% reported the same for information relevant to patients. | The research findings converge to address the need for educators and administrators in the UAE to develop guidelines concerning its safe use and proactively integrate e-professionalism into their respective curriculum. |
| **28** | *Jiang, Z., et al. 2021* | 59 | Twelve tips for teaching medical students online under COVID-19 | Perspective | NA | NA | Provide 12 tips to highlight strategies intended to help on-site medical classes moving completely online under the pandemic. | Collected ‘best practices’ reports from 40 medical schools in China that were submitted to the National Centre for Health Professions Education Development. Experts’ review-to-summary cycle was used to finalize the best practices in teaching medical students online that can benefit peer institutions most, under the unprecedented circumstances of the COVID-19 outbreak. | Refer to Figure 1. All Twelve Tips for Teaching Medical Students Online under COVID-19. | The 12 tips presented offer-specific strategies to optimize teaching medical students online under COVID-19, specifically highlighting the tech-based pedagogy, counselling, motivation, and ethics, as well as the assessment and modification. Learning experiences shared by pilot medical schools and customized properly are instructive to ensure a successful transition to e-learning. |
| **29** | *Joshi, A. R., et al. 2020* | 128 | Effect of nationwide lockdown due to coronavirus disease-19 pandemic on daily activities and study pattern of the 1st MBBS students. | Quantitative | 5.5 | NA | The study was planned in, first MBBS (Phase I) students to analyze study pattern, perception toward various aspects of online teaching and daily activities during nationwide lockdown period. | This study was undertaken among the 1st MBBS (Phase I, Block III) student volunteers in a local medical college. After approval by the Institutional Ethical Committee, a Google Form was designed, specifically based on the daily routine, perception toward various aspects of online teaching, time spent on studying and study pattern of Phase I students. The data was represented as mean±standard deviation. The responses were tabulated in percentage | About 93.9% of the students were primarily engaged in studies and 79.2% of students were involved in exercise at home. About 84% of students were satisfied with online teaching sessions. About 70.5% and 59.7% of the students felt an increased sleep duration and screen time on mobile respectively, while only 26.2% of the students felt that their study time was increased. They were involved in various health related activities and hobbies and had quality time with parents. | This lockdown had a positive effect toward the optimistic thought processes of the students while engaging in family bonding and revisiting hobbies for a short duration. It is seen to be effective and helpful to the students having online lectures. |
| **30** | *Kang, Y. J. & D. H. Kim 2021* | 77 | Pre-clerkship students' perception and learning behavior of online classes during coronavirus disease 2019 pandemic | Quantitative | 9.5 | NA | This study evaluates the feasibility and effectiveness of this approach, aiming to identify the advantages and dis- advantages of online-sCP from the medical students’ perspectives. | To develop the survey items, 20 pre-clerkship students were interviewed about their learning experiences in the first semester of 2020. A total of 425 pre-clerkship students have participated in the survey, which comprised four parts (general experience, learning strategies, important features, and overall satisfaction). | Before classes began, students generally had “neutral expectations” about online classes (2.90 out of 5). At the end of the semester, overall satisfaction with the curriculum was moderate (3.04 out of 5). Premedical students reported lower scores in “daily study hours” and “regular lifestyle” and higher scores in “experience of playing recorded lectures solely for an attendance check” (p<0.001). In addition, first-year premedical students not only showed significantly lower utilization of learning strategies compared to other grades (p<0.001). | Although pre-clerkship students mostly shared similar perceptions and behaviors in their learning regardless of grade level, some characteristics were either more prominent in premedical students on the whole or limited to first-year premedical students. |
| **31** | *H. Kasai et al. 2021* | 98 | Alternative approaches for clinical clerkship during the COVID-19 pandemic: online simulated clinical practice for inpatients and outpatients-A mixed method | Qualitative and Quantitative | 13 | 16 | The sEHR was reviewed by medical students and subsequently discussed with a supervising physician using an online meeting system | Among 118 fifth-year medical students in 2020, only medical students who participated in the online-sCP for the Respiratory unit and General Medicine were included. A group of 10 or 11 medical students underwent a four-week training program as members of a medical team of doctors and residents until February 28, 2020. Between May and July 2020, a four-week online-sCP was conducted in the Respiratory unit and General Medicine departments. | Even though there was an attempt to access online professionalism, students still cited coverage of this as a disadvantage. | Online-sCP with sEHR, e-PBL, and online-VMI could be useful in learning some of the clinical skills acquired through CC. These methods can be implemented with limited preparation and resources. Furthermore, these methods have the potential to serve as efficient and sustainable educational methods that can be used in exreme situations. |
| **32** | *D. Kay; M. Pasarica 2019* |  | Using technology to increase student (and faculty satisfaction with) engagement in medical education | Qualitative | NA | 21 | The study designed and deployed four (2 preclinical, 2 clinical) lifestyle medicine sessions to explore the impact on, as well as our own subjective satisfaction with, students’ levels of substantive engagement. | *Preclinical: nonmandatory sessions. We deployed a preclinical traditional type (PCTT) and a preclinical technology-enhanced type (PCTE) session to second-year medical students in two subsequent academic years, as nonmandatory sessions with the same learning objectives. PCTT was a face-to-face session, where groups of six students were tasked with solving a clinical scenario (24) on paper. Each group submitted its response to the course discussion board. Each response was projected on a large screen, visible to all students, when groups presented their submission. Students in attendance were invited to provide feedback to the submissions from other groups, and the faculty member provided “real-time” feedback.* | Paper discusses the benefits of sometimes turning off cameras to facilitate professionalism | we elected to introduce low-risk course formats that utilized technologies like chat, discussion boards, and icons, such as the “thumbs-up” feature, and correctly predicted these would have a positive impact on students’ engagement behaviors. We optimized the value of when and where students accessed a session to explore how these variables would impact student engagement behaviors and were happy to see an increase in both the level and quality of the student interactions. It is our hope that this report will encourage and inform faculty who are navigating this ever- widening gap between faculty expectations and students’ actual demonstrations of engagement in medical education. |
| **33** | *C. Kenyon; L. Lingard 2011* | 94 | What would socrates think? Undergraduate medical students' experience in a distributed medical education network | Conference Abstract | NA | NA | The study explored the experience of medical students studying in a distributed network using VC. | Using a grounded theory approach and theoretical sampling, semi-structured interviews were conducted with 11 students studying at a distributed medical education site. We employed constant comparative analysis to identify emerging themes. A focus group was conducted to verify and refine the themes following preliminary data analysis. | Implied: Issues due to a lack of professionalism | Although VC can be an acceptable alternative to face-to-face lectures, studying in a remote site can impact student learning in unpredicted ways. Medical schools need to be cognizant of the strengths and weaknesses of VC in developing distributed medical education networks. |
| **34** | *R. Khalil et al. 2020* | 89 | The sudden transition to synchronized online learning during the COVID-19 pandemic in Saudi Arabia: a qualitative study exploring medical students' perspectives | Qualitative | NA | 19 | the present study aimed to explore undergraduate medical students' perceptions regarding the effectiveness of synchronized online learning at Unaizah College of Medicine and Medical Sciences, Qassim University, Saudi Arabia. | A qualitative study was conducted using virtual focus group discussions synchronously with the help of a discussion guide consisting of seven open-ended questions. Overall, 60 medical students were recruited using a maximum variation sampling technique; these students then participated in eight focus group discussions. All interviews were recorded, transcribed verbatim, and analyzed for thematic contents using the standard (Mayring, Kiger. M. E. and Braun.V) | **Technological Issues** Difficulties experienced due to technological hindrances of internet connectivity and poor utility of online tools.  eg. “Slow internet connectivity and communication software failure **Instructor issues** “Sometimes instructor’s voice was not clear and they didn’t use appropriate explanatory tools  “Some instructors were not in a habit of checking their microphones before starting their lectures, **Barriers from Behavioural challenges** Barriers in adoption of online learning influenced by the individual personality characteristics **(implied) Possible reasons for lack of professionalism** Multiple lectures in one day Duration of lectures was too long **Student claiming that non-verbal communication is important (implied HENCE MUST ENFORCE CAM)** In my opinion, non-verbal communication like eye contact with the instructor is essential to establish learning process.  **Lack of conducive environment** “Most of the times, I did not find a suitable place at home for taking my online classes and I felt like environment is not suitable at home for attending online lectures.” | This represents significant and promising potential for the future of medical education. The principles of the online learning model and learning outcomes should be rigorously and regularly evaluated to monitor its effectiveness. |
| **35** | *K. Knie et al. 2020* | 135 | To zoom or not to zoom - the training of communicative competencies in times of Covid 19 at Witten/Herdecke University illustrated by the example of sharing information"" | Qualitative | NA | 12 | Since October 2018, a longitudinal communication curriculum for medical students has been implemented at Witten/Herdecke University. In the summer semester 2020, the concept for the 4(th) preclinical semester included a practical training on "sharing information", which consisted of three two-hour face-to-face sessions with simulated patients | In two digital sessions, the students then were able to practice discharge interviews and discussions about risk communication illustrated by the example of screening methods for cancer prevention. In the first zoom session, students practiced in role-plays among themselves. In the second zoom session, they practiced with SP. The evaluation results revealed that 76% of the responding students considered working with the e-learning module as a good preparation for the interviews. | **Citing the Problem** almost half of them confirmed that using Zoom significantly impaired the atmosphere (47%) | The students explicitly appreciated working with SP. From the teachers' perspective, some specific aspects of successful communication were difficult to reflect on, e.g. non-verbal communication. The use of e-learning as a preparation for practical exercises has proven successful and will be continued in the future. |
| **36** | *G. Korkmaz; C. Toraman 2021* | 141 | Exploring Medical Students' Readiness for E-Learning and Knowledge Sharing Behaviors in Emergency Remote Learning Environments during COVID-19 | Quantitative | NA | 17 | This study aims to examine the medical students' readiness for e-learning and knowledge sharing behaviors in online learning environments during COVID-19, and to analyze the relationship between these variables. | The study has been designed according to correlational research methodology. The data were collected using "Knowledge Sharing Behavior in Online Learning Environments Scale" and "E-Learning Readiness Self-Assessment Instrument". 725 medical students participated in the study. Students' readiness for e-learning and knowledge sharing behaviors were analyzed using descriptive statistics, and the relationship between the variables was modeled using multivariate regression equation. | Student isolation was cited as a short term implication of lacking a standard for online professionalism | The results revealed that the students with high ability to use computer, mobile phone and tablet PC have higher readiness level for e-learning in terms of technology access, technical skills, online relationships, motivation, online skills, importance of success. |
| **37** | *M. D. Krasowski et al. 2021* | 69 | Teaching Pathology in an Integrated Preclinical Medical School Curriculum and Adaptations to COVID-19 Restrictions | Descriptive | NA | NA | The study describes the experience of adapting preclinical medical school courses within an integrated curriculum to virtual instruction | NA | **Marking attendance** Student attendance (participation) captured via Report function of Zoom **Asking questions** Students were directed to bring questions forward via the Chat function | The author discusses the challenges and successes of the switch to virtual instruction and of teaching pathology content within an integrated medical school curriculum. |
| **38** | *C. Krebs et al. 2021* | 140 | Ethics behind technology-enhanced medical education and the effects of the COVID-19 pandemic | Opinion Piece | NA | NA | NA | NA | Educators must remain committed and be persistent in learning how to engage new technologies in order to prevent the loss of ethical principles and professionalism, as well as interpersonal relationships and mentoring, thus avoiding isolation, the production of incompetent healthcare professionals and unfit pedagogics. | NA |
| **39** | *Kuroda. N, et al. 2022* | 1231 | Predicting the effectiveness of the online clinical clerkship curriculum: Development of a multivariate prediction model and validation study | Quantitative | 12 | NA | This study aimed to develop and externally validate a prognostic score that represents students’ satisfaction and motivation, and can predict the effectiveness of the online clinical clerkship curriculum. | The study consisted of two parts. First, we collected the survey responses for the derivation dataset. Through derivation analysis, we developed a scoring model for the online curriculum to predict medical students’ satisfaction and motivation. We then collected the survey responses for the validation dataset. Developed using the derivation dataset, we assessed our predictive model’s prediction performance via validation analysis. | The model’s prediction performance was high despite not incorporating individual demographic information.  With the proposed scoring system, educators will be able to both predict the effectiveness of and improve their online clinical clerkships. Our instrument will be of great help to educators in building a better online clinical clerkship curriculum. | We successfully developed a scoring model to predict the effectiveness of the online clinical clerkship curriculum, based on students’ satisfaction and motivation. Our scoring model will accurately predict and improve the online clinical clerkship curriculum’s effectiveness. |
| **40** | *I. R. Lee et al. 2021* | 118 | Changes in undergraduate medical education due to COVID-19: a systematic review | Qualitative | NA | 11 | This study aims to provide medical educators with insights into the current status and prospects of undergraduate medical education, which has been affected by the COVID-19 pandemic. | We conducted a database search of PubMed, Embase, and ERIC and identified articles on COVID-19-related undergraduate medical education. We independently reviewed titles and abstracts and extracted data on the geographic location of the study, area of specialty, phase in medical school (preclinical year, clerkship year, etc.), type of paper, and the main content of the study. | Citing of problem: Need for institutional strategies, or there is an urgent need to implement new modalities apart from virtual education Poor technical skills  Inadequate infrastructure | This review summarized the essential changes in undergraduate medical education worldwide and reflected on the various teaching methods adopted by medical schools. In preparation for the post-COVID era, a comprehensive online curriculum and evaluation tools are needed, which require the development of necessary infrastructure and adequate resources. Education aimed at helping students be more socially aware and responsible as medical professionals must be promoted. |
| **41** | *H. T. T. Leung et al. 2021* | 60 | Teaching psychiatry to medical students in the time of COVID-19: experiences from UK medical schools | Qualitative | NA | 11 | The study aimed to understand the impact of psychiatric education due to the COVID pandemic | Education leads for undergraduate psychiatry in UK medical schools completed questionnaires on adaptations made to undergraduate psychiatry education, their impact and what lessons could be learnt for the future. | The need for setting expectations (address problem) | Despite the extraordinary efforts of education leads to maintain undergraduate psychiatry education, the pandemic may affect the development of students' professional competencies and recruitment into psychiatry. Individual clinicians, trusts and medical and foundation schools have much to offer, and need to work with students to replace what has been lost during the pandemic. |
| **42** | *Lin, E., et al. 2021* | 75 | Comparison of In-Person and Telesimulation for Critical Care Training during the COVID-19 Pandemic | Quantitative | 12.5 | NA | This was a prospective observational cohort study assessing the impact of an in-person versus remote simulation course on fourth-year medical students from February to April 2021 at the University of California San Diego School of Medi- cine. | Precourse and postcourse surveys were constructed (Appendix E2 and E3). The questions were reviewed by a group of physicians to assess validity of self-reported items and assess for suitability and com- pleteness based on previously published recommendations (17). As questions were reviewed by the study design team and based on previously validated methodology, no pilot validation study was per- formed. The surveys were distributed immediately before and after the course using an online secure resource (Qual- trics.com) in accordance with guidelines on proper web-based administration (18). All of the postcourse surveys were com- pleted within 1 week. | Overall, when polled about whether simulation was a good way to learn critical care skills in a safe learning environment, students strongly agreed with an average score of 5.0 out of 5.0 in in-person learners and 4.9 in remote learners. Regardless of the training modal- ity, the students had a positive experience with the simulation course, ranking it 9.6 out of 10 (9.9 in in-person vs. 9.3 in telesi- mulation; P = 0.06). All of the students recommended the addition of this simulation course to their medical curriculum. | In conclusion, remote learning with telesimulation is feasible, and this teaching modality can be effectively incorporated into a simulation curriculum for medical students. Clinician educators should select a telesimulation technique that matches their resources and may have to adjust their learning objectives to this digitized format. Furthermore, they should consider the use of checklists or other tools to optimize telesimulation debriefing if their telesimulation model includes remote learners. Educators should be aware that a telesimulation-based simulation course may not be as effective for remote learners as active in-person participants but still suggests the ability to improve provider readiness across technical and cognitive domains when approaching critical care cases |
| **43** | *T. Loda et al. 2020* | 126 | Medical education in times of COVID-19: German students' expectations - A cross-sectional study | Quantitative | 10 | NA | In this study, they investigated the teaching- and learning-related stressors and expectations of medical students in Germany during the COVID-19 pandemic. | A cross-sectional survey was distributed online to undergraduate medical students at medical faculties in Germany. Students answered questions about COVID-19 and teaching (on a 7-point Likert scale from 0 ("not at all") to 6 ("completely")) and completed mental well-being measurements, including the State-Trait Anxiety Inventory (STAI), the Generalised Anxiety Disorder scale (GAD-7) and the Perceived Health Questionnaire (PHQ-9). Descriptive data analysis, a t-test and Pearson correlations were performed to process the data. | Students were dissatisfied with the teaching style, pace, which explained suboptimal participation | Medical students seem to be aware of the COVID-19 pandemic and its consequences for academic and healthcare contexts. They also seem to think that their teachers will enhance their digital competencies during the pandemic. Therefore, faculties of medicine need to rapidly and adequately digitalise their approaches to teaching. |
| **44** | *McFadden, S,. et al. 2022* | 117 | Academic, clinical and personal experiences of undergraduate healthcare students during the COVID-19 pandemic: A prospective cohort study | Quantitative and Qualitative | 9 | 20 | A prospective cohort design was implemented to meet the aim of the study. The study was set in Ulster University Northern, which is geographically spread across 4 campuses within Northern Ireland. The University has interprofessional training incorporated across all health courses with placements carried out in local University teaching hospitals. | Students completed surveys at three time- points across the academic year: Time 1 (T1) in October, Time 2 (T2) in December 2020 and Time 3 (T3) in April 2021. Quantitative survey data was downloaded from Qualtrics into SPSS Version 25 for analysis. A cross sectional analysis was used to generate descriptive statistics to provide a snapshot of student’s experiences at focal timepoints. Some results included two or three timepoints to enable comparisons over time; other results were specific to each timepoint due to variations in the survey questions. Results are presented as valid percentages, excluding missing data where applicable. Qualitative data was also downloaded from Qualtrics into text format, which was then coded and thematically analysed using content analysis. Data from the survey was checked by the research group and consensus reached through discussion. | Although the majority of students had access to a laptop suitable for academic work and mostly rated platforms for remote learning as excellent or average, challenges in relation to having access to reliable fast broadband, a desk suitable for academic work and a quiet/adequately sized working space impacted upon the experience of remote learning for many students.  Lower use of Blackboard chat rooms (Approximately 32% of participants had not used chat rooms at Time 1 and Time 2) may have been a missed opportunity to maxi- mise engagement with Generation Z students, viewed as ‘digital natives’ [20] who are familiar with the use of digital participatory spaces [21]. Nevertheless, going forward they expressed a preference for a more flexible approach to their academic experiences using a blended approach of synchronous and asynchronous course delivery and remote and university based learning challenging universities, academics and students to maximise engagement with digital technologies. | Given the potential for future and continued pandemic restrictions, as well as an expressed preference for a blended approach to academic learning going forward, universities should consider how learning programmes can meet the needs of students in terms of the develop- ment of their practical skills, the ability to access support, manage their screen time and work- load as well as being able to maintain engagement and provide opportunities for peer interactions. |
| **45** | *M. D. Mehta et al. 2020* | 107 | Analysis of online classroom vs physical classroom learning methods according to 2nd year medical college students’ perception | Abstract Only | NA | NA | Teaching learning methodology have impact on knowledge, attitude and communications skills of students. Due to advanced technology, online classroom is possible over physical classroom. Purpose of the study is to know its effectiveness by comparing with physical classroom | Total 120 students participated from 2nd MBBS, medical college after giving informed consent. They filled pre-validated Google questionnaires. Data was collected and analysed further. | Full text couldn't be found; but students felt distracted more easily, with the majority of students agreeing that if networking tools are set up properly, online learning may just be immersive enough | Online classroom is not permanent solution for teaching learning methodology in medical education system. In situation like COVID-19, online video lecture with efficient networking tools may provide feel of face-to-face learning. |
| **46** | *E. Moradi 2020* | 23 | Netiquette and its application in virtual learning: a vital necessity in medical universities during the Coronavirus pandemic | Opinion Piece | NA | NA | NA | NA | The word "Netiquette" comes from the two words "Net" meaning network and the Internet and "Etiketa" , which refers to a set of rules of behavior and social habits and (the right way to socialize with others) | Therefore, it is suggested that in the universities of medical sciences, a system for teaching and counseling the etiquette be designed for both components of the educational system, namely faculty members and students, to acquaint them with this important category from possible disorders in communication skills. |
| **47** | *A. Morice et al. 2020* | 127 | Virtual versus traditional classroom on facial traumatology learning: Evaluation of medical student's knowledge acquisition and satisfaction | Qualitative | NA | 16 | The objectives of this study were: to compare student's knowledge acquisition and satisfaction between virtual and traditional face-to-face classroom and to determine potential factors that may correlate with the level of knowledge acquisition following both type of courses. | Our study included fifth-year medical students attending the course of facial traumatology, who voluntarily participated either in virtual or traditional classroom. Students' knowledge acquisition was evaluated through a multiple-choice questions test. A second questionnaire aimed to determine factors potentially correlated with students' knowledge acquisition and to evaluate their satisfaction. Results of questionnaire n(o) 1 were compared between virtual and traditional classroom. Additionally, a correlation study was performed between the results of questionnaire n(o) 1 and the factors listed in questionnaire n(o) 2. | Cited lessons are too long, overall time commitment needed to finish online courses too demanding | Considering there is no noticeable difference in knowledge acquisition between virtual and traditional classroom, we will continue virtual classroom in parallel with traditional classroom and extend it to the other courses of our field. |
| **48** | *S. Mubeen et al. 2020* | 136 | Analysis of the e-learning educational atmosphere during covid 19 pandemic: Empirical evidence from medical universities of urban pakistan | Quantitative | 5.5 | NA | To analyze the level of satisfaction of medical students towards the existing e-learning educational environment and to determine its effectiveness enhancing learning outcomes. | This study was conducted at the Online survey was conducted using google forms to collect data from MBBS and BDS students enrolled in various public medical universities of Karachi and who have attended online classes during COVID-19 pandemic from April to September 2020. Materials and Methods: Structured questionnaire using a 5-point Likert scale was adopted for the study with reliable Cronbach's alpha coefficient (0.85). Non-probability purposive sampling technique was used for selecting the participants. | Students cited reduced motivation to study during lockdown, due to time management issues | The survey revealed positive attitude of medical students towards e-learning. However, clinical exposure of students is compromised which is a major challenge. Depending on the requirement of specific courses, adopting a hybrid approach involving some combination of e-learning and practical exposure seems more effective. |
| **49** | *S. Muflih et al. 2021* | 86 | Online learning for undergraduate health professional education during COVID-19: Jordanian medical students' attitudes and perceptions | Quantitative | 14 | NA | The aim of this study was to assess students' attitudes towards online learning as well as the perceived preparedness and barriers. | A descriptive, cross-sectional, correlational web-based survey design was used to recruit eligible participants from five Jordanian government universities. A Facebook-based campaign and snowball sampling approach were used to recruit potential survey participants. | Could be attributed to an unstable Internet connection, a lack of motivation, a lack of instructions, a home environment that is not prepared for online learning, Internet access fees, online learning being boring, and the time commitment of online learning. Similar findings were reported in previous studies | The majority of students had mixed feelings about online learning and were largely supportive of conventional classroom learning. Students were pessimistic about their chances of learning professional skills and core competencies online. More research is required to determine whether students are ready and able to make greater use of online education in order to access high-quality learning opportunities. |
| **50** | *E. Nordmann et al. 2020* | 61 | Ten simple rules for supporting a temporary online pivot in higher education | Opinion Piece | NA | NA | NA | NA | Establish the Guidelines to Students | NA |
| **51** | *A. T. Oladipo et al. 2020* | 121 | Challenges with medical education in Nigeria in the COVID-19 era | Descriptive | NA | NA | In this essay, we take a look at medical education in Nigeria, its challenges and progression in the COVID-19 era. We also take a look at the effect of the pandemic on learning and the subsequent interventions introduced to mitigate it. | NA | Highlighted lack of adequate technical infrastructure for staff and students | This therein has caused loss of valuable time and a change in the calendar of the school year, making it uncertain as to when the next set of qualified medical professionals will emerge in Nigeria. |
| **52** | *F. Otaki et al. 2021* | 138 | Introducing the 4Ps Model of Transitioning to Distance Learning: A convergent mixed methods study conducted during the COVID-19 pandemic | Qualitative | NA | 21 | This study aimed to shed light on the distance learning experiences of medical students and their instructors at the Mohammed Bin Rashid University of Medicine and Health Sciences (MBRU) in Dubai, United Arab Emirates | A convergent mixed methods approach was utilized. Qualitative and quantitative data was collected using a survey of closed-ended followed by open-ended questions. | Students did complain about not meeting learning objectives | The qualitative analysis led to developing the 4Ps Model of Transitioning to Distance Learning, which encapsulates four interrelated themes. It would be helpful to leverage the lessons learned to tailor blended medical programs with a reasonable mélange of experiences. The study also contributes to the mixed methods research by showcasing a means of adapting it to evaluate critical situations reliably and rapidly. |
| **53** | *N. Paul et al. 2020* | 97 | Integration of Technology in Medical Education on Primary Care During the COVID-19 Pandemic: Students' Viewpoint | Descriptive | NA | NA | The study explores how medical students have benefited from these virtual tutor groups and how similar small-group online teaching opportunities can add value to the medical curriculum in the future. | NA | Students complained about no clear explanation given to students | The role of technology in health care is undoubtedly expanding at a rapid pace, and this is especially true in the COVID-19 era, where noncontact solutions to health care needs have become essential. As final-year medical students, we believe that it is crucial that we are equipped to adapt to different formats of remote working and to address any associated challenges that may present in the future. There have been several technological adaptations to global medical curricula during the past months, such as the transition of physical lectures to web-based formats; however, many other aspects of medical teaching have been paused until face-to-face teaching can safely be resumed. We believe that by integrating simulated remote consultations through VTGs, students can continue to develop their communication skills and clinical acumen, and this should be considered as a permanent inclusion in the post–COVID-19 medical curricula. |
| **54** | *R. M. Peloso et al. 2020* | 108 | Notes from the Field: Concerns of Health-Related Higher Education Students in Brazil Pertaining to Distance Learning During the Coronavirus Pandemic | Qualitative | NA | 14 | This survey aimed to assess the concerns of students of health-related higher education in Brazil regarding distance learning during the coronavirus pandemic. | A Google Forms anonymous questionnaire was sent by WhatsApp Messenger to students at a private university. Seven hundred and four students answered the questionnaire (566 female, 138 male, mean age = 23.09 years), reflecting approximately a third of the students in health-related disciplines | Self-Discipline from students lacking; reported easy distractions | Health-related higher education private institutions in Brazil should focus on reassessing and prioritizing their policies and protocols and include a detailed plan for the future. |
| **55** | *O. Popa-Velea et al 2021* | 95 | Teaching Style, Coping Strategies, Stress and Social Support: Associations to the Medical Students' Perception of Learning during the SARS-CoV-2 Pandemic | Quantitative | 14 | NA | This study assessed the learning perception of undergraduate medical students on three types of teaching (classical/online/hybrid), in relation to coping strategies, stress, and social support, in the context of the SARS-CoV-2 pandemic. | 201 students (48 men, 153 women; mean age = 22.900, SD = 2.830) participated in the study. They answered a Multidimensional Scale of Perceived Social Support, a Brief Cope Scale, a Student-life Stress Inventory, a visual analog scale for usefulness, and a survey collecting their perceptions about learning across teaching types. | Evidence citing behavioural disengagement (loss of attention) Evidence of lack of good communication with profs and peers. | These data may contribute to the strategic growth and refinement of web-based teaching methods in medical universities. |
| **56** | *Riedel, M,. et al. 2021* | 129 | Experiences with alternative online lectures in medical education in obstetrics and gynecology during the COVID‐19 pandemic—possible efficient and student‐orientated models for the future? | Quantitative | 10 | NA | The main goal of our study was to evaluate the extent to which a concise transfer of knowledge from a clini- cal expert to students—as is traditionally carried out via lectures—could be substituted by potentially more-time- efficient and student-orientated online-learning resources | The 61-item questionnaire was disseminated following the multiple-choice exam at the end of the obstetrics and gynecology course in the 2020 summer semester. Study participation was voluntary and independent of the exam. All students provided written consent for participation. The questionnaire included 31 items with 5-point Likert- scale ratings that the participants used to indicate their agreement or disagreement with the statement for each item (1 = *strongly disagree (–)*, 2 = *disagree (-)*, 3 = *neither agree nor disagree (-/* +*)*, 4 = *agree (* +*)*, 5 = *strongly agree (*+ +*)*). Other questions were either dichotomous or classi- fication questions. | The fact that most lectures take place early in the morning and that they require continuous presence during the semester does not align with the preferences of a large proportion of our students. Bati et. al investigated reasons for non-attendance of lectures among Turkish medical-science students and found that sleepless- ness and teaching inefficiency of lectures stood out  A major finding of our analysis was that direct interaction with the lecturer or the ability to ask questions were only relevant for a minority our students. By contrast, socializ- ing with fellow students and friends was evaluated as being equally important during lectures. Likewise, Shah et al. reported that among American osteopathic medical students, a large proportion of time spent during lectures was used to study for other classes or was spent on social media or reading emails [32]. The benefits of compulsory attendance with or without being physically present in a lecture hall are, therefore, questionable. Our students differentiated between the requirements of small-group learning in seminars that relied on direct interaction among the attendees and the pas- sive delivery of information in lectures. There was no sig- nificant difference between students’ appraisal of screencasts and online webinars. Nonetheless, flexibility may also require a higher degree of learning discipline. A curriculum with more e-learning offerings and without a strict timetable could be more prone to neglect students with weaker learning skills and self-motivation | Our data demonstrate that the traditional teaching format of face-to-face lectures does not meet the flexibility demanded by today’s generation of medical students. E-learning, on the contrary, is practical and appreciated by our students and leads to equivalent test results compared with regular teaching methods at a German university hospital. Apart from possible setbacks in overcoming COVID-19 in the near future, various e-learning formats might be feasible tools for making medical education more student-orientated. |
| **57** | *P. N. Rocha; N. A. A. de Castro 2014* | 64 | Opinions of students from a Brazilian medical school regarding online professionalism | Quantitative | 14 | NA | To determine the frequency with which students from a Brazilian Medical School come across ten given examples of unprofessional online behavior by medical students or physicians, and gather the opinions of participants regarding the appropriateness of these behaviors. | A cross-sectional survey of 350 students from the Medical School of Bahia, Brazil. Only those who had a profile in social media were included in the final analyses. | Only 13.5 % reported having discussions about online professionalism during ethics classes. Among the examples of unprofessional online behaviors investigated in this study, the most commonly observed by medical students were use of foul language, pictures in which they are wearing bathing suits and those related to alcohol consumption. These behaviors drew much less rejection than violation of patients’ privacy. | Medical students are witnessing a high frequency of unprofessional online behavior by their peers and physicians. Most investigated behaviors were considered inappropriate, especially if carried out by physicians. Participants were not able to recognize the publication of pictures of hospitalized children or neonates in social media as cases of violation of patients’ privacy. Further studies are needed to determine if an academic curriculum that fosters online professionalism will change this scenario. |
| **58** | *Saddik, B., et al. 2020* | 112 | Increased Levels of Anxiety Among Medical and Non-Medical University Students During the COVID-19 Pandemic in the United Arab Emirates | Quantitative | 10 | NA | The COVID-19 pandemic is likely to increase anxiety levels within the community and in particular medical students who are already considered psychologically vulnerable groups. Since the COVID-19 outbreak, no study has yet estimated the effect of this pandemic on university students in the UAE or its impact on the psychological wellbeing of medical students. | Surveyed 1485 medical (comprising medical and dental) and non-medical university students across 4 emirates within the UAE. Used an online platform to assess knowledge, sources of information, changes in hygienic behavior, perceptions of fear and worry and anxiety levels using the generalized anxiety disorder 7 (GAD-7) scale. The GAD-7 score was measured at three time points: during hospital visits for medical/dental students, before the introduction of online learning and after online learning for all students | Majority of students demonstrated high levels of knowledge and utilized reliable sources of information. Non-medical students exercised higher compliance with social restrictions, while medical students practiced better hand hygiene. Almost half of students reported anxiety levels ranging from mild to severe with females reporting higher anxiety scores during hospital visits (OR=2.02, 95% CI, 1.41 to 2.91) and medical students reporting lower anxiety levels in comparison to dental students (OR=0.61, 95% CI, 0.45 to 0.84). Medical students reported higher levels of anxiety during their clinical rotations which decreased with the introduction of online learning, yet, non-medical students’ anxiety levels increased with online learning | Study provides important information on the initial response and anxiety levels in university students across the UAE during the COVID-19 pandemic. Findings from study can be used to support the development of effective screening strategies and interventions to build psychological resilience among university students during the COVID19 pandemic or any other public health emergencies in the future. |
| **59** | *Sadoski, M. & C. C. Colenda 2010* | 119 | The Texas A&M experience with class size and campus expansion: evaluation of first year using distance learning and on-site curriculum delivery | Quantitative | 10.5 | NA | Doubled class size and double number of fully matriculating branch campuses to answer calls for an increased physician workforce. Article describes the 1st full year's experience with expansion. Distance learning was a key part of the experience. | After extensive planning and pilot study, 1st-year students were fully matriculated at 1 of 2 campuses. Year-end comparisons were made on students’ achievement and a satisfaction questionnaire. | No statistically significant differences were found on any achievement measures and only several questionnaire items | Expansion from the 2+2 system to 2 fully-matriculating campuses with a larger student body was successful during its 1st year. Some persistent problems and planned solutions are reported. |
| **60** | *Said, J. T. & A. W. Schwartz 2021* | 63 | Remote Medical Education: Adapting Kern’s Curriculum Design to Tele-teaching | Descriptive | NA | NA | COVID-19 has necessitated a rapid shift to the remote delivery of medical education | Presenting a timely collection of tips, techniques, and strategies for the facilitation of remote teaching sessions and modification of curriculum design, assessment, and evaluation. | Step through Kern’s six-step curriculum design, recommending to (1) consider session necessity and a variety of teaching models; (2) inform your session with surveys and polls; (3) keep session endpoints consistent; (4) make the most of technology and translate in-person strategies to virtual forms; (5) engage with individual learners and eliminate distractions; and (6) consider online methods of assessment and evaluation methods. | Instil confidence and improve the virtual classroom, allowing for the broad delivery of high-quality remote medical education to all learners affected by COVID-19 and beyond. |
| **61** | *Seymour-Walsh, A. E., et al. 2020* | 76 | Practical approaches to pedagogically rich online tutorials in health professions education | Commentary | NA | NA | Strategies for learning activity design and tutor training are proposed to equip course designers and educators to support health professions education remotely, through the synchronous, online small group. | This article considers established learning theories, with examples of how they might apply to small group HPE activities during COVID-19 and beyond. It will propose pedagogically informed and engaging online small group learning to address the current needs of HPE, and promote improved future access to rich online learning for the diaspora of RRR clinicians and students. | **Introduction** In an online group, the educator’s ability to develop relationships with the learners may be hampered; therefore, the identification of individuals’ motivations, difficulties and experiences may not be so apparent. As with online lectures, an online tutorial group can be perceived by students as a passive learning activity, which can occur with the camera disabled and microphone muted, while other distracting home tasks are performed. **Conclusion** Students will focus on processing what is in the screen, and neglect the opportunity to analyse, problem-solve, explore and create learning together. **Course designers can promote an interactive environment to support communities of inquiry in an online tutorial by..... (important points) -** encouraging participants to upload a photo of themselves as an avatar, so that if video feeds fail, the group still feels that they are communicating with people rather than with a system. **Discussion** Learners may not all be familiar with participating in an online facilitated tutorial, and the inevitable technological and interaction barriers may be wearing. A tutor who acknowledges these, navigates flexible workarounds and encourages the group to share their thoughts as valuable group offerings, will promote a smooth transition to an unfamiliar yet safe learning environment. | May herald a new era of renewed teaching opportunities for rural, regional and remote educators, and richer options of engagement for learners and clinicians based beyond urban communities, into the future |
| **62** | *Sharma, D. & S. Bhaskar 2020* | 92 | Addressing the Covid-19 Burden on Medical Education and Training: The Role of Telemedicine and Tele-Education During and Beyond the Pandemic | Systematic Scoping Review | NA | 10 | Aim to critically evaluate the impact of COVID-19 on medical education, training and medical students; and to make targeted recommendations to maintain continuity and support mental health, well-being and education needs of affected students. | Relevant literature was identified via PubMed and Medline review, including original, opinion and perspective articles, topic reviews, official national medical associations/bodies and societal guidelines and media sources. The PubMed/Medline search was performed using the keywords “Medical Students,” “COVID-19” and “Medical Education” until July 31, 2020. The PICO template, with the population (medical students), intervention (COVID19), comparator (standard medical education pre-COVID19) and outcome (impact on medical students/education and changes adopted due to COVID-19), was used. The literature was examined to critically analyse existing structural and systemic challenges of medical education, with an emphasis on the use of technologies such as telemedicine or remote education, and formulate a synthesis on the impact of COVID-19 on medical education, students and training. Appropriate articles relevant to COVID-19 were included in this synthesis. Medical students who are especially vulnerable, such as those with pre-existing mental illness, disadvantaged backgrounds, overseas medical students and those in under resourced settings are considered and impact of COVID-19 on these subgroups are presented. | Medical students are at increased risk of mental or psychological disorders, with a significantly higher prevalence of depression, depressive symptoms and suicidal ideations relative to the general population (8) (Table 1). Disruptions in traditional medical education and training due to COVID-19 have increased risk of poor mental health among medical students worldwide (Table 2) (8–16). The mental health burden could be exacerbated in those with pre-existing mental illness (4, 42). Concerns around inadequate skill development due to suspension of hospital placements, ambiguity around future prospects and subsequent financial implications have been reported. Poor health behaviors, sleep deprivation during COVID-19 and pre-existing chronic diseases among medical students could adversely affect physical and mental health (29), with cardiovascular disease, diabetes, obesity and chronic neurological comorbidities associated with increased risk of hospitalization and severe illness due to COVID-19. | Medical students vulnerable during the current pandemic, with subgroups of medical students from specific backgrounds more impacted. Targeted support for these subgroups, and students overall, is warranted. COVID-19 has exposed systemic issues within our healthcare and education systems. Recognizing these issues and developing strategies to combat them is pivotal to our response to an infection outbreak in the future. |
| **63** | *Sharma, N., et al. 2021* | 87 | Perception towards online classes during covid-19 among mbbs and bds students in a medical college of nepal: A descriptive cross-sectional study | Descriptive cross-sectional | 8 | NA | Aim of study to find out the perception towards online classes during COVID-19 lockdown period among MBBS and BDS students at a medical college of Nepal | Descriptive cross-sectional study carried out at Universal College of Medical Sciences and Teaching Hospital among first and second year Bachelor in Medicine, Bachelor of Surgery and Bachelor in dental surgery students from 1st June 2020 to 30th August 2020. Ethical approval was taken from Institutional Review Committee of Universal College of Medical Sciences and Teaching Hospital (IRC UCMS, Ref: UCMS/IRC/025/20). Convenient sampling method was used. Semi-structured questionnaire was used. Statistical Package for Social Sciences 22 was used for analysis and frequency and percentage was calculated. | One hundred fifty six (73.93%) students were enjoying online learning only to some extent, 135 (63.98%) felt online class not equally effective as face-to-face teaching. The students had disturbance during online classes as internet disturbance 168 (79.60%), and electricity problem 47 (22.3%). Similarly, many students 155 (73.50%) felt external disturbance, headache 26 (12.3%), and eye strain 26 (12.3% | Most of the students suffered from disturbances during online classes probably because of internet and electricity problem. When compulsory to conduct online classes, students felt that not more than three online classes per day should be conducted to avoid eye strain and headache. |
| **64** | *Shehata, M. H., et al. 2021* | 62 | A toolbox for conducting an online OSCE. | Descriptive | NA | NA | Highlights the lessons learned and the tools used to run the online OSCE at the College of Medicine and Medical Sciences, Arabian Gulf University (CMMS-AGU) using Zoom™️. | Planning and preparation phase in which situation analysis, aligning stakeholders, mobilizing resources, creating a shared vision, and ownership of the exam project take place. For successful implementation of examinations, detailed plans are needed including manpower, timings, number of stations and detailed description of the steps of the examination process. Provided a set of guiding questions for proper decision making related to online clinical exams. Implementation Phase in which piloting is very useful to apply improvements to the original plan and to outline the needed capacity building of the participating staff. Gave a detailed description of the guiding documents, means of communication and features of ZOOM that were used. Evaluation phase provides a guide for evaluating the process and outcome, including a list of key performance indicators. | **Establishing Guidelines** aligning stakeholders, mobilizing resources, creating a shared vision, and ownership of the exam project take place. A document that consists of the crucial steps to be adhered to during the exams to maintain standardization, prevent technical failures, uphold highest levels of integrity, and achieve confidentiality. **Checking Identity** (Zoom rooms where students are hosted by an invigilator and asked to bring out their photo ID so that invigilators make sure of their identity) **Proper Planning** A core team that has diverse skills like computer skills, communication skills etc. is a pivotal success factor for online assessments. This necessitates robust training and capacity building in either technical or organizational tasks. Such training can be held either through online meetings, face to face meetings, and on-the-job training during the mocks. | The process, although doable, needs a lot of investment in manpower and preparation including technical specifications, documents preparation, involvement of staff with diverse job descriptions, training of people, and continuous quality improvement. This toolbox is intended to guide the process and to make the outcome as valid and reliable as possible. This method can be used to assess clinical skills thereby saving more time and space for physical examination skills during face to face exams. |
| **65** | *Singal, A., et al. 2021* | 101 | Anatomy education of medical and dental students during COVID-19 pandemic: a reality check. | Quantitative | 11.5 | NA | To understand the visible and invisible potential challenges being faced by the 1st year medical and dental students while attending digital anatomy classes. | Conducted on 81st year medical and dental students who were admitted to their respective college in August 2019 and were willing to participate in the study. A multiple choice close-ended questionnaire regarding their opinion on virtual classes was designed and feedback was taken from the students | Majority (65%) of the students agreed that they missed their traditional anatomy learning i.e., dissection courses, face to face lectures and interaction with mentors. The students strongly felt the lack of confidence and difficulty in the topics completed without dissections, models, microscopic slides and other modalities. 83% felt lack of proper gadgets, high-band width and strong internet connections, a potential barrier in their digital learning. Lack of self-motivation was felt by 69% students | The current situation of anatomy education is not intentional, and is not the long term silver bullet solution for a visual subject like anatomy. Though learners face a lot of challenges, however, a shift to online must be supported at this time of health crisis. As the digital learning may go for indefinite period, the feedback of students may be helpful for relevant and timely modifications in digital anatomy education |
| **66** | *Singh, R., et al. 2021* | 96 | Perception towards online teaching-learning in medical education among medical students during COVID-19 outbreak in Nepal: A descriptive cross-sectional study. | Descriptive Cross-sectional | 11 | NA | To describe the perception of medical students towards online teaching-learning introduced during the COVID-19 outbreak in Nepal. | An online survey using a descriptive cross-sectional study design was carried out among 515 undergraduate medical students currently enrolled in medical colleges in Nepal. A semi-structured questionnaire in Google form was utilized to collect data. The link of the Google form was sent to the potential respondents through email and social media. Descriptive statistics, including frequency, percentage, mean, and standard deviation were used to analyze data in SPSS vs20. Ethical approval was sought from Nepal Health Research Council to conduct this study, and digital informed consent was taken from study respondents | The overall score of perception of online teaching-learning was 17.61±7.19, which indicated many problems in this method of teaching-learning. The mean score of perception of online teaching-learning was found to be different across sex, location of enrolled medical colleges, having a personal electronic device, having an internet connection at residence, having separate room/space for attending online classes, and self-rated computer skills. Moreover, only 28 (5.4%) of respondents had perceived online teaching-learning as a better method of delivering content of medical curricula. | Surveyed medical students in Nepal were found to perceive many problems in online teaching-learning. Moreover, management and faculty members need to take the necessary measures for enhancing the online teaching-learning quality. |
| **67** | *Slivkoff, M. D., et al. 2021* | 130 | First-Year Medical Student Experiences Adjusting to the Immediate Aftermath of COVID-19 | Qualitative | NA | 15 | Describe the emergent mid-course transition that took place for first-year medical students at one US medical school due to COVID-19 and an evaluation of students’ adjustments to both new methods of learning and new ways of living due to social distancing restrictions | s On the final day of the course, students were sent surveys which assessed how they adjusted academically and personally to the campus shutdown | Students cited inability to manage time, because of lack of schedule The response rate was 137/159 (86%). Students’ learning adjustments took into account changes to spaces and daily routine, their cohabitants, need for accountability, new learning resources, and anxiety. Most students were concerned about public health, the economy, and health of family and loved ones; fewer were concerned about their professional futures, restrictions on personal freedoms, and own health. Most students adjusted personally by connecting more with family, entertainment and sleep, and studying less. While a large majority of students made changes to connecting with friends and physical activity, students did not adjust uniformly | Contextualizing students’ academic and personal experiences in the immediate aftermath of the COVID-19 crisis in the USA will inform and encourage studies at other institutions and learning settings, and a realization that each student’s experience must be appreciated when providing the support they need for academic and personal development |
| **68** | *Stoehr, F., et al. 2021* | 12 | How COVID-19 kick-started online learning in medical education-The DigiMed study | Quantitative | 12.5 | NA | Aim to evaluate students’ attitudes to online learning to provide a broad scientific basis to guide future development of medical education | Overall, 3286 medical students from 12 different countries participated in this cross-sectional, web-based study investigating various aspects of online learning in medical education. On a 7-point Likert scale, participants rated the online learning situation during the pandemic at their medical schools, technical and social aspects, and the current and future role of online learning in medical education. | The majority of medical schools managed the rapid switch to online learning (78%) and most students were satisfied with the quantity (67%) and quality (62%) of the courses. Online learning provided greater flexibility (84%) and led to unchanged or even higher attendance of courses (70%). Possible downsides included motivational problems (42%), insufficient possibilities for interaction with fellow students (67%) and thus the risk of social isolation (64%). The vast majority felt comfortable using the software solutions (80%). Most were convinced that medical education lags behind current capabilities regarding online learning (78%) and estimated the proportion of online learning before the pandemic at only 14%. In order to improve the current curriculum, they wish for a more balanced ratio with at least 40% of online teaching compared to on-site teaching. | This study demonstrates the positive attitude of medical students towards online learning. Furthermore, it reveals a considerable discrepancy between what students demand and what the curriculum offers. Thus, the COVID-19 pandemic might be the long-awaited catalyst for a new “online era” in medical education. |
| **69** | *Stojan, J., et al. 2021* | 133 | Online learning developments in undergraduate medical education in response to the COVID-19 pandemic: A BEME systematic review: BEME Guide No. 69 | Systematic Scoping Review | 12 | NA | This systematic review investigated the pivot to online learning for nonclinical undergraduate medical education (UGME) activities and explored descriptions of educational offerings deployed, their impact, and lessons learned. | The authors systematically searched four online databases and conducted a manual electronic search of MedEdPublish up to December 21, 2020. Two authors independently screened titles, abstracts and full texts, performed data extraction and assessed risk of bias. A third author resolved discrepancies. Findings were reported in accordance with the STORIES (STructured apprOach to the Reporting in healthcare education of Evidence Synthesis) statement and BEME guidance. | Fifty-six articles were included. The majority (n¼41) described the rapid transition of existing offerings to online formats, whereas fewer (n¼15) described novel activities. The majority (n¼27) included a combination of synchronous and asynchronous components. Didactics (n¼40) and small groups (n¼26) were the most common instructional methods. Teachers largely integrated technology to replace and amplify rather than transform learning, though learner engagement was often interactive. Thematic analysis revealed unique challenges of online learning, as well as exemplary practices. The quality of study designs and reporting was modest, with underpinning theory at highest risk of bias. Virtually all studies (n¼54) assessed reaction/satisfaction, fewer than half (n¼23) assessed changes in attitudes, knowledge or skills, and none assessed behavioral, organizational or patient outcomes. | UGME educators successfully transitioned face-to-face instructional methods online and implemented novel solutions during the COVID-19 pandemic. Although technology’s potential to transform teaching is not yet fully realized, the use of synchronous and asynchronous formats encouraged virtual engagement, while offering flexible, self-directed learning. As we transition from emergency remote learning to a post-pandemic world, educators must underpin new developments with theory, report additional outcomes and provide details that support replication. |
| **70** | *Sundarasamy, V. G., et al. 2020* | 104 | Psychological impact of COVID-19 on medical college students. | Quantitative | 10 | NA | The present study aims to conduct an online survey to investigate the mental health status of students from a medical college in India. | From March to August 2020, a cross-sectional survey was conducted among 470 medical college students from the coastal area in India using standard questionnaires measuring adverse psychological outcomes. Multivariate regression was used to examine the determinants of adverse psychological outcomes. | Health difficulties faced by students during the lockdown period were measured. Alteration in their sleep pattern was prevalent in this sample of medical college students, and 65.3 % indicated positive screens for significant reduction in their attendance and concentration level. Due to the long-lasting pandemic situation and onerous measures such as lockdown and stay-at-home orders, the COVID-19 pandemic brings negative impacts on higher education. The findings of our study highlight the urgent need to develop interventions and preventive strategies to address the mental health of college students. | To ensure the continuous involvement of students in educational processes, the universities should initiate all-inclusive online-based educational programs to reach out the students living in remote areas with or without devices in association with internet-service providers by providing scholarship or student loan. Furthermore, parents should be encouraged, by providing pandemic response and recovery support from the government, to create a friendly and positive family environment for university students without imposing pressure on the future academic and working career. |
| **71** | *Taggar, J., et al. 2021* | 48 | Clinical placements in General Practice: concepts and considerations of implementing remote virtual placements in the COVID world | Descriptive | NA | NA | This article describes some of the approaches that enable remote (home) virtual patient encounters in Primary Care for medical students. | These are categorised as methods that a) enable remote access into GP clinical systems, b) enable remote access into individual patient consultations and c) enable an observational-only experience | Key considerations are highlighted to enable safe and effective implementation of remote virtual consultations, along with the advantages and disadvantages of each method. These include patient consent, confidentiality, data sharing and protection, professionalism, student agreements and data gathering templates. | Further evaluation and iterative refinement of models is required to ensure that approaches to virtual consultations remain adequate for clinical learning. |
| **72** | *Taluja, M. K., et al. 2021* | 103 | A study of impact of coronavirus disease-19 pandemic on behavior and teaching of medical students | Quantitative | 10 | NA | To assess the advantages and disadvantages of online teaching as well as behavioral changes in medical students as a result of lockdown due to COVID-19. | Online questionnaire-based study was conducted in 112 MBBS students. The questionnaire comprised demographics, addiction history, behavioral changes, sleep disturbances, e-learning through online classes, and relaxation techniques used to combat the behavioral changes associated with COVID-19 lockdown. | In lockdown period, the arrogance/ irritation increased in 42% of students, 48.2% observed more anger, frustration, lack of energy, loneliness, and 50.9% were more anxious for little things, whereas 65.2% of students worried about future. | Concluded that there is increase in level of stress and anxiety among MBBS students |
| **73** | *Thind, A. S., et al. 2021* | 91 | Impact of the COVID-19 pandemic on Caribbean Medical Students: A cross-sectional study. | Quantitative | 12 | NA | To further explore the impact of COVID-19 on medical training in the Caribbean, | A cross sectional designed survey was constructed and made available for completion to numerous 2nd, 3rd and 4th year medical students between the dates of Feb 2nd, 2021, and April 1st, 2021. | Students reported being less time efficient and paying less attention during online lectures. Many students reported having their Comp, Step 1, Clinical rotations, and research projects delayed because of the lockdown. Most students ranked 10/10 on anxiety and depression scores during the lockdown. | The changes made to the Caribbean Medical school system due to the Pandemic have shown to be less effective than previous methods of instruction. It is crucial for programs to consider this for the future to optimize learning and continue to produce highly trained medical professionals even in the face of adversity. |
| **74** | *Toraman, C. 2021* | 116 | Medical Students' Curiosity, Exploration and Engagement Levels in Online Learning Environments during COVID-19 | Quantitative | 10 | NA | This study has been designed according to the type of correlational research in which the researcher measures two variables and assesses the statistical relationship between those variables (Fraenkel, Wallen, & Hyun, 2012). The study focuses on the examination of the levels of medical students’ curiosity and exploration, classroom engagement and commitment to online learning environment during the COVID-19 pandemic as well as to analyze the relationship between these variables. | In this study, the data were collected through three different tools.  Trait Curiosity and Exploration Inventory, Student Engagement Scale in Online Learning Environment, Classroom Engagement for University Students.  In this way, data were obtained from 618 students in total out of 994 students (according to 2020-2021 Academic Year) who study at the Faculty of Medicine, Çanakkale Onsekiz Mart University. The participation rate is 62%, which indicates that the data could not be obtained from the entire population. Therefore, purposive sampling method was adopted. | According to these findings, it can be stated that medical students often try to adapt to the online classes and want to benefit from this environment. These findings correlate with several studies in the literature (Rajab, Gazal & Alkattan, 2020; Zheng & Zhang, 2020). However, when the items with low mean were examined, it was also determined that the students had problems with concentration in the online environment, they do not feel excited about online classes and do not consider learning online as fun, and the activities in the online were not interesting. Similarly, Ravi, R. C. (2020) stated that neither students nor educators feel excited because of the quality of activities during online classes.  On the other hand, the students stated that the classes were not fun, they did not orally participate in the classes, they were not willing to answer the questions. These findings are similar to the findings of the research conducted by Bock et al. (2020). It can be concluded from these findings that the students do not often participate in the class discussions.  Flexibility, as a subfactor of curiosity and exploration, is a significant positive predictor of affective, behavioral and cognitive engagement (p<.05). When flexibility increases, affective, behavioral and cognitive engagement also increase. Acceptance uncertainty, as a subfactor of curiosity and exploration, is a positive predictor for behavioral engagement but a negative predictor for cognitive engagement (p<.05). When acceptance uncertainty increases, behavioral engagement increases whereas cognitive engagement decreases. | To improve students’ engagement in online learning in general, the educators should use different platforms and tools to provide a more participatory learning environment, and these tools should aim to enhance learning through collaboration. In addition, different methods and techniques of teaching should be utilized to support the students learning. |
| **75** | *Torda, A. 2020* | 124 | How COVID-19 has pushed us into a medical education revolution | Opinion | NA | NA | Highlight some ways in which the COVID-19 pandemic has changed medical education, particularly pushing us into online delivery for our pre-clinical years. | - | - | - |
| **76** | *Triemstra, J. D., et al. 2021* | 71 | Impact of the COVID-19 Pandemic on the Clinical Learning Environment: Addressing Identified Gaps and Seizing Opportunities | Descriptive | NA | NA | Health professions educators and learners from multiple institutions and specialties discuss the gaps and weaknesses exposed, opportunities revealed, and strategies developed for optimizing the CLE in the post–COVID-19 world. | - | Virtual spaces should be crafted to make sure that all learners have equal access to high-speed, secure internet; professional backgrounds for video meetings; and devices with adequate processing speeds and security features. To maintain compliance with the Health Insurance Portability and Accountability Act of 1996, 39physical spaces within the CLE will need to accommodate UME and GME learners who do not have access to private work spaces in their personal residences to maintain equity in health professions education for all | - |
| **77** | *Vala, N. H., et al. 2020* | 111 | Study of anxiety, stress, and depression level among medical students during covid-19 pandemic phase in jamnagar city. | Quantitative | 8 | NA | This study was conducted to evaluate mental status and prevalence of anxiety, stress, and depression among medical students during coronavirus disease (COVID)-19 phase. | This study was conducted in 250 1st -year MBBS students of Shri M. P. Shah Government Medical College, Jamnagar, after getting approval from the Institutional Ethical Committee. Pre-structured questionnaire-based DASS21 scale was used to conduct this study through Google form after obtaining written consent from the students. | In our study, we found that prevalence of anxiety, stress, and depression in 1st -year medical students was 17.20%, 15.60%, and 10.80%, respectively. | In our study, we found that anxiety, depression, and stress are present in medical students. In COVID-19 phase, students are not attending medical colleges and all the teaching process is online. Even at home, medical students are suffering from anxiety, depression, and stress. Early evaluation and intervention should be done to reduce anxiety, depression, and stress in medical students. |
| **78** | *Wang, J., et al. 2021* | 106 | Perceived Stress Among Chinese Medical Students Engaging in Online Learning in Light of COVID-19. | Quantitative | 12 | NA | This study will comprehensively evaluate the distribution of stressors of medical students and explore the personal and environmental predictors of PS during the epidemic. | An online survey was conducted among medical students (n=369) from three medical universities in western China who engaged in online learning. A stress process conceptual framework was formed to explore the influencing factors of PS. The survey items contained four sections: (a) the potential stressors derived from academic, psychosocial and health-related demands; coping resources such as (b) online learning environment support and (c) personal resilience, including online learning behavior and individual characteristics; and (d) PS, perception of imbalanced demands and coping resources | The mean PS score was 17.39 (SD=4.58), and over four-fifths (82.3%) of the students had moderate to high levels of stress. The average item scores of academic, psychosocial and health-related stressors were 2.72 (SD=0.55), 2.31 (SD=0.55) and 2.07 (SD=0.50), respectively. Gender, grade, psychosocial stressors, health-related stressors, specific online learning behavior (persistence, attitude and flexibility), and the online learning environment (teaching, social and cognitive presence) were predictors of PS. | Our results specify that a reduction in psychological and health-related stressor stimulation, specific online learning behavior promotion, and well-established online learning environment support could be considered essential for alleviating the negative impacts of COVID-19 on the psychosocial health of medical undergraduates. |
| **79** | *Wilkie, V. 2009* | 66 | Online learning in primary care: the importance of e-professionalism | Opinion | NA | NA | - | - | - | - |
| **80** | *Wolniczak, E., et al. 2020* | 99 | Course of macroscopic anatomy in Magdeburg under pandemic conditions | Qualitative | NA | 12 | : The Covid-19 pandemic has created major challenges for university teaching. At the beginning of the summer semester 2020, teaching at the Medical Faculty in Magdeburg was almost completely online. Also the course in macroscopic anatomy had to be replaced by virtual e-learning offers | Videos and photo presentations of the preparation steps and structures to be displayed were made available online. The reactions of the students showed very quickly that the three-dimensionality, the independent preparation and the haptics of the object to be studied make up a large part of this subject | Virtual e-learning offerings are a useful supplement to, but not a substitute for, active dissecting on body donors. By changing the course offerings in compliance with hygiene and distance rules, we were able to offer a classroom course again during the semester, which was expressly welcomed by the students | The majority of students clearly state that a virtual dissection course cannot replace the real dissection course. The entire college was able to gather a great deal of experience in order to be able to react adequately and provide the students with the best possible training under pandemic conditions. Based on these experiences (teaching and logistics), the teaching can be designed for the coming winter semester; in classroom, hybrid or online courses |
| **81** | *Zis, P., et al. 2021* | 103 | Medical Studies during the COVID-19 Pandemic: The Impact of Digital Learning on Medical Students' Burnout and Mental Health. | Quantitative | 11 | NA | The aim of this ecological study was to investigate what the impact of digital learning due to the COVID-19 pandemic was on the burnout and overall mental health (MH) of medical students | : An anonymous questionnaire was administered to all 189 eligible candidates before and during the COVID-19 pandemic. Mental health was assessed via the MH domain of the 36-item Short Form Health Survey (SF-36) and burnout with the Maslach Burnout Inventory—Student Survey (MBI-SS) | : The overall response rate was 81.5%. The overall burnout prevalence did not differ significantly between the two periods (pre-COVID-19 18.1% vs. COVID-19 18.2%). However, the burnout prevalence dropped significantly in year 4 (pre-COVID-19 40.7% vs. COVID-19 16.7%, p = 0.011), whereas it increased significantly in year 6 (pre-COVID-19 27.6% vs. COVID-19 50%, p = 0.01). When looking at each MBI-SS dimension separately, we found that emotional exhaustion decreased significantly in year 4 but increased in year 6, and cynicism increased in all years. The overall MH deteriorated significantly between the two periods (pre-COVID-19 58.8 ± 21.6 vs. COVID-19 48.3 ± 23, p < 0.001). | Digital learning in medical studies carries significant risks. Not only does the MH deteriorate, but cynicism levels also increase. Emotional exhaustion was found to increase particularly in final year students, who struggle with the lack of clinical experience just before they start working as qualified junior doctors. |
| **82** | *Zureick, A. H., et al. 2018* | 93 | The interrupted learner: How distractions during live and video lectures influence learning outcomes. | Quantitative | 11 | NA | New instructional technologies have been increasingly incorporated into the medical school learning environment, including lecture video recordings as a substitute for live lecture attendance. The literature presents varying conclusions regarding how this alternative experience impacts students’ academic success. | Here, three cohorts of first-year medical students (N5439 respondents, 86.6% response rate) were surveyed in greater detail regarding lecture attendance and video usage, focusing on study behaviors that may influence histology learning outcomes. | Students who reported always attending lectures or viewing lecture videos had higher average histology scores than students who employed an inconsistent strategy (i.e., mixing live attendance and video lectures). Several behaviors were negatively associated with histology performance. Students who engaged in “non-lecture activities” (e.g., social media use), students who reported being interrupted while watching the lecture video, or feeling sleepy/losing focus had lower scores than their counterparts not engaging in these behaviors. | This study suggests that interruptions and distractions during medical learning activities—whether live or recorded—can have an important impact on learning outcomes. |
| **83** | H. Moses Murdock,  John C. Penner,  Stephenie Le, and Saman Nematollahi | 82 | Virtual Morning Report During COVID‐19: A Novel Model for Case‐Based Teaching Conferences | Descriptive | NA | NA | The article described the result of the implementation of a virtual morning report in a hospital due to social distancing measures imposed during the initial stages of the COVID-19 Pandemic. | NA | **SUPPORTIVE MEASURES THAT CAN PROMOTE NETIQUETTE**: By asking specific questions of the chat, facilitators can engage passive participants and elicit a robust conversation that includes differential diagnoses, problem representations, and diagnostic reasoning. Ongoing priorities include minimizing inaccurate information and encouraging participants to not focus too heavily on naming the final diagnosis as quickly as possible.   Fostering a supportive learning environment on a virtual platform requires special attention. Open-access platforms are vulnerable to unwanted participants. On multiple occasions, hackers invaded VMRs and shared explicit comments and images, known as “Zoom-bombing.” Hosting password-protected Zoom sessions shared through a moderated email listserv ensured VMR’s integrity. Additionally, a large number of participants makes it difficult to ensure all exchanges are supportive. Indeed, comments from passive participants were occasionally critical or disrespectful. After sharing at the start of each VMR the expectation that participants communicate with respect and assigning a senior faculty member to moderate chat content, negative comments fell. | The article supports VMR as a viable model for virtual case-based teaching conferences. Advantages include adaptability during physical distancing, accessibility via asynchronous viewing, and the opportunity for multi-institutional participation. Future work includes evaluating the impact of VMR on trainee’s clinical reasoning practices and exploring the utility of integrating VMR into clinical reasoning curricula. |
| **84** | *The University of Hong Kong LKS Faculty of Medicine* | 67 | E-learning Rules and Etiquette | Guidelines | NA | NA | NA | NA | NA | NA |
| **85** | *Faculty of Health, Keele University* | 83 | Netiquette Guidelines | Guidelines | NA | NA | NA | NA | NA | NA |
| **86** | *NYU Grossman School of Medicine* | 70 | Student Handbook | Guidelines | NA | NA | NA | NA | NA | NA |
| **87** | *University of Rochester School of Medicine and Dentistry* | 73 | Student Handbook | Guidelines | NA | NA | NA | NA | NA | NA |
| **88** | *Indiana University School of Medicine* | 84 | Medical Student Social Media and Online Activity Policy | Guidelines | NA | NA | NA | NA | NA | NA |
| **89** | *University of Washington School of Medicine* | 72 | Standards of Conduct and Professional Behavior Policy | Guidelines | NA | NA | NA | NA | NA | NA |
| **90** | *OHSU School of Medicine* | 85 | Medical Student Handbook | Guidelines | NA | NA | NA | NA | NA | NA |
